# Analysis of spinal and muscle pathology in transgenic mice overexpressing wild-type and ALS-linked mutant MATR3

**DOI:** 10.1186/s40478-018-0631-0

**Published:** 2018-12-19

**Authors:** Christina Moloney, Sruti Rayaprolu, John Howard, Susan Fromholt, Hilda Brown, Matt Collins, Mariela Cabrera, Colin Duffy, Zoe Siemienski, Dave Miller, David R. Borchelt, Jada Lewis

**Affiliations:** 1 Center for Translational Research in Neurodegenerative Disease, University of Florida, Gainesville, Florida, USA; 2 Department of Neuroscience, University of Florida, Gainesville, Florida, USA; 3 McKnight Brain Institute, Department of Neuroscience, University of Florida, Gainesville, Florida, USA

**Keywords:** MATR3, Transgenic mouse model, ALS, Distal myopathy

## Abstract

Mutations in *MATR3* have been associated with amyotrophic lateral sclerosis (ALS) as well as a form of distal myopathy termed vocal cord pharyngeal distal myopathy (VCPDM). To begin to understand how mutations in *MATR3* may cause disease, here we provide initial characterization of transgenic (Tg) mice expressing human wild-type (WT) MATR3 (MATR3^WT^) and ALS-mutant F115C MATR3 (MATR3^F115C^) proteins under the control of the mouse prion promoter (MoPrP). For each construct, we established multiple independent lines of mice that stably transmitted the transgene. Unexpectedly, for all stably-transmitting lines examined, *MATR3* transgenic mRNA expression was more robust in muscle, with minimal expression in spinal cord. The levels of transgenic mRNA in muscle did not differ between mice from our lead MATR3^F115C^ line and lead MATR3^WT^ line, but mice from the lead MATR3^F115C^ line had significantly higher levels of MATR3 protein in muscle over the lead MATR3^WT^ line. Mice from the three independent, established lines of MATR3^F115C^ mice developed weakness in both fore- and hind-limbs as early as < 1 months of age; whereas, MATR3^WT^ mice aged to > 20 months were not overtly distinguishable from non-transgenic (NT) littermates based on basic motor phenotype. Muscle of both MATR3^WT^ and MATR3^F115C^ mice showed vacuoles by 2 months of age which worsened by ~ 10 months, but vacuolation was noticeably more severe in MATR3^F115C^ mice. Overall, our results indicate that increasing the levels of MATR3 in muscle can cause pathologic changes associated with myopathy, with MATR3^F115C^ expression causing overt muscle atrophy and a profound motor phenotype. The findings suggest that analysis of muscle pathology in individuals harboring ALS-linked MATR3 mutations should be routinely considered.

**Electronic supplementary material:**

The online version of this article (10.1186/s40478-018-0631-0) contains supplementary material, which is available to authorized users.

## Introduction

Amyotrophic lateral sclerosis (ALS) is a progressive neurodegenerative disease affecting both upper and lower motor neurons leading to progressive paresis and paralysis, with death occurring between three to five years from the diagnosis [18]. The majority of ALS cases appear sporadic (sALS), with around 10% of cases having a clear genetic link between family members (fALS) [18]. Superoxide dismutase 1 (*SOD1*) was the first gene to have mutations identified as causing ALS [17]. Since the late 2000s, there have been over a dozen genes in which mutations have been identified as causative for ALS, including Tar DNA binding protein (*TARDBP*) [21, 27], fused in sarcoma (*FUS*) [10, 22], and the GGGGCC hexanucleotide repeat expansion in chromosome 9 open reading frame 72 (*C9orf72*) [4, 16], with matrin 3 (*MATR3*) being one of the more recently discovered genes to have disease causing mutations [7].

Mutant MATR3 (S85C) had been previously associated with an autosomal dominant distal myopathy with vocal cord and pharyngeal weakness (VCPDM) [5, 19]; however, in 2014, three different mutations (F115C, P154S, or T622A) in *MATR3* were identified in cases of both fALS and sALS [7]. Patients harboring the S85C mutation in *MATR3* initially present with myopathy in the muscles of the hands and feet [5, 19] and have a slowly progressing disease that leads to respiratory failure and death after 15 years of illness. The S85C *MATR3* mutation is associated with muscle pathology including rounded fibers, rimmed subsarcolemmal vacuoles and internalized nuclei [2, 5, 26]. In contrast, patients carrying the F115C mutation in *MATR3* develop dysarthria, dysphagia, weakness and variable degrees of dementia, with a typical course of 5 years before death [7]. Spinal cord tissue from F115C patients displayed intense staining of MATR3 within the motor neuron nuclei as well as occasional cytoplasmic immunostaining of MATR3 in neurons compared to spinal cords from control subjects [7]. Subsequent to the identification of ALS-linked *MATR3* mutations, Johnson and colleagues reclassified the original VCPDM S85C cases as ALS [7]; however, there have now been additional families with the S85C mutation that have been diagnosed with VCPDM, without indication of ALS [2, 12, 13, 19, 26], highlighting our lack of understanding of which disease(s) *MATR3* mutations cause.

In an effort to determine the natural regulation of MATR3 in the body, we previously found that murine MATR3 expression decreased with age from postnatal day 1 through 2 months of age in the central nervous system (CNS) [15]. Murine MATR3 was low in the spinal cord compared to several other regions of the CNS [15]. Additionally, MATR3 was difficult to detect in the skeletal muscle of adult mice [15]. It was interesting that both the spinal cord and muscle had such low levels of MATR3, given that they are pathologically affected in ALS or distal myopathy [15].

Given the low expression of MATR3 in the skeletal muscle and spinal cord of normal adult mice and that MATR3 gene trap mice were not reported to display a phenotype akin to ALS or distal myopathy [14], we sought to determine whether increasing expression of WT or mutant MATR3 through transgenesis would produce ALS or myopathic phenotypes. We drove expression of human MATR3 utilizing the mouse prion promoter (MoPrP), a promoter that is active in the spinal cord and muscle [3]. Our original intent was to determine if expression of ALS-linked F115C MATR3 or VCPDM- and ALS-linked S85C MATR3 in Tg mice would lead to distinct or overlapping phenotypes and pathologies. We attempted to control for the effects of Tg expression by also creating mice expressing WT human MATR3. Unexpectedly, transgene expression in these animals was highest in muscle with minimal expression in spinal cord, with the levels of MATR3^F115C^ being significantly higher than that of mice expressing MATR3^WT^ at the protein level in our lead lines. MATR3^S85C^ mice did not robustly express MATR3 protein in either muscle or spinal cord compared to NT mice and were excluded from further study.

Both the MATR3^WT^ and MATR3^F115C^ mice developed myopathic changes that progressed with age from small vacuoles to large vacuolated fibers, rounded fibers, and fibers with internalized nuclei. The MATR3^F115C^ mice developed obvious muscle weakness with a variable age of onset, between ~ 0.9 and 10 months of age, depending on the line. By contrast, MATR3^WT^ mice typically did not show obvious weakness through 20 months of age. Data from lead MATR3^WT^ and MATR3^F115C^ lines indicate that increasing the levels of MATR3 protein in muscle produces myopathic changes. These mice provide new in vivo tools that can be used to dissect the role of MATR3 in muscle degeneration.

## Materials and methods

### Animal husbandry

Mice were kept on a 12-h light/12-h dark cycle with ad libitum access to food and water. All procedures were approved by the Institutional Animal Care and Use Committee of the University of Florida and complied with the National Institute of Health’s “Guide for the Care and Use of Laboratory Animals.”

### Generation of Tg animals

Tg mice expressing either WT or mutant human MATR3 were generated by cloning the human *MATR3* cDNA to drive expression of the full-length MATR3 protein, the predominant species in murine CNS [15]. pCMV-Sport6 (ThermoScientific, catalog #MHS6278–202757255) was used as the template to amplify human WT *MATR3* cDNA by PCR using primers 5′ attctctagggtcgaccaccATGtccaagtcattccagcag 3′ and 5′ tcactctagggtcgacTTAagtttccttcttctgtctg 3′. This was cloned into the *Sal* I site of a modified pEF-BOS vector using the InFusion HD cloning kit, (Clontech, catalog #639649). No 5′ or 3′ untranslated region of the human MATR3 mRNA was included in the cDNA gene used to produce the mice; the untranslated sequences were derived from the MoPrP vector [3]. The upstream PCR primer includes an optimized Kozak sequence of CCACC immediately before the start codon. The pEF-BOS-MATR3 vector was digested with *Sal* I, the *MATR3* cDNA was gel purified, and ligated into the *Xho* I site of the MoPrP vector, which was then microinjected into fertilized FVB/NJ oocytes (Jackson Laboratory) and implanted into pseudopregnant females. The F115C and S85C mutations in the *MATR3* cDNA were created using the QuikChange II XL Site-directed Mutagenesis kit (Agilent, catalog #200522) and the mutant construct for each was prepared using the same procedure as the WT *MATR3* cDNA, as mentioned above. Seven MATR3^WT^ founders were mated with FVB/NCr mice (Charles River), six of which transmitted the transgene (Lines 8, 9, 59, 60, 1554, 1563). Five MATR3^F115C^ founders (70, 72, 1573, 1576, 1579) were mated with FVB/NCr mice (Charles River) to generate hemizygous F1 offspring, all of which transmitted the transgene. Five MATR3^S85C^ founders (209, 212, 213, 257, 275) were mated with FVB/NCr mice (Charles River), all of which transmitted the transgene. Genotyping was performed on tail biopsy DNA from mice using the following primers: (PrP-sense) 5’ GGGACTATGTGGACTGATGTCGG 3′, (PrP-antisense) 5’ CCAAGCCTAGACCACGAGAATGC 3′, and (*MATR3-*sense) 5’ AGCAAGAGCTTGGACGTGTG 3′. Mice were designated as Tg by the presence of a band approximately 1100 bp corresponding to the transgene and a 750 bp band produced by the endogenous *PrP* allele, while NT contained only the 750 bp endogenous *PrP* band.

### Confirmation of *MATR3* allele in transgenic offspring

We verified the specific human *MATR3* allele by either sequencing or restriction digest of a human *MATR3* amplicon from tail biopsy DNA from the majority of the experimental mice (Additional file 1: Table S1, sequencing, asterisk; restriction digest, ^§^). The MATR3^S85C^ mice were discontinued before this verification process. In most cases, we confirmed the allele of *MATR3* present in the Tg mice using PCR to amplify *MATR3* transgene cDNA with the following primers: (sense) 5’ ATTCTCTAGG*GTCGAC*CACCATGTCCAAGTCTTCCAGCAG 3′ [complementary to 5′ elements of cDNA-*Xho*I cloning site italicized and start codon underlined] and (antisense) 5’ CGTTGAAAATCCATGATGTCAACATCTCTGCTAGTTTCCACTCT 3′ [complementary to sequences spanning the junction of exons 4 and 5]; resulting in an 1200 bp PCR product. The PCR products were purified using a Monarch PCR & DNA cleanup kit (New England BioLabs catalog #T1030S) and then the purified PCR products were sent to GenScript for sequencing (Additional file 1: Table S1, asterisk) using the following primer which would allow for unambiguous identification of the sequences encoding both S85 and F115 positions: (antisense) 5’ CCATAACTCAAGGTAGAGCCTTCTTCAGTTC 3′.

In some cases, restriction digestion of the PCR amplicon (generated as above) was used instead of sequencing (Additional file 1: Table S1, ^§^) to confirm the presence or absence of the F115C mutation. 10 μL of the purified PCR product was then digested with *Pvu*II for 15 min at 37 °C and run on a 1% agarose gel. Since the F115C mutation creates a *Pvu*II site, MATR3^WT^ mice have one band at 1.2 kb, and MATR3^F115C^ mice have bands at 345 and 850 bp.

### Phenotyping

Mice were phenotyped into two groups: mild-to-moderate (MM) or severe (S) phenotype based on cage behavior and escape extension. The MM phenotype displayed as moderate muscle weakness with no change to the escape extension, while the S phenotype appeared strikingly impaired when ambulating and an abnormal escape extension. In many cases, UF Animal Care Staff technicians initially identified animals that were developing a phenotype without knowledge of the genotype and informed the lab. In all cases, an observer unblinded to genotype assessed the levels of weakness, changes in body mass, and muscle size in phenotyping the affected mice. In some cases, an additional observer (J.L) validated phenotyping by blindly identifying mice with motor phenotypes.

### Harvesting and tissue collection

Mice were euthanized by cervical dislocation or anesthetized with isoflurane, followed by exsanguination and perfusion with PBS. The right gastrocnemius, right quadriceps, and a small portion of the cervical spinal cord were snap frozen on dry ice and stored at − 80 °C until preparation of lysate or RNA. The remainder of the spinal cord and left hindlimb were dissected and immersion fixed in 10% formalin. After 24 h, fixed spinal cord and hindlimb muscles were transferred into PBS. All tissues were stored in PBS at 4 °C until processing.

### Northern blotting

RNA was isolated from quadriceps and spinal cord using Trizol (Invitrogen, catalog #15596026) per manufacturer protocol. A DNA probe was made by digesting the PrP vector with *Eco*RI and *Xho*I, isolating a 450 bp fragment, which encompassed sequences in the 3′ untranslated segment of the transgene construct, and purifying it with Monarch Gel Extraction kit (New England BioLabs catalog #T1020S). This probe for the blots was labeled with α-^32^P-dCTP using Rediprime II DNA Labeling System (GE Healthcare Amersham, catalog #RPN1633) and purified using ProbeQuant™ G-50 Micro Columns (GE Healthcare, catalog #28903408), both per manufacturer protocol. 5 μg of RNA was incubated with 3x volume of loading dye (360 μL formamide, 80 μL 10x MOPS buffer, 120 μL 37% formaldehyde, 50 μL glycerol, 10 μL 10% BPB) for 2–3 min at 100 °C, then cooled on ice. 1 μg of 0.5 mg/mL ethidium bromide was added to each sample, and then was resolved on the gel (1% agarose, 2% formaldehyde/MOPS buffer) at 120 V. The gel was soaked in de-ionized H_2_O for 40–60 min with three changes to remove formaldehyde and transferred to GreenScreen Plus nylon membrane (Perkin-Elmer, catalog #NEF976) by capillary action overnight using 10X SSC buffer. Next, crosslinking to membrane occurred using the autocrosslink function of the Stratalinker (UVP, model CL-1000). The following was completed at 65 °C. In the prehybridization step, the blot was incubated in hybridization buffer [1% BSA, (fraction V); 1 mM EDTA, pH 8; 0.5 M sodium phosphate, pH 7.2; 7% SDS; de-ionized H_2_O] for two hours. The buffer was discarded, and the blot was hybridized in hybridization buffer with boiled probe (0.5 × 10^6^ cpm/mL) for ~ 18 h. The blot was rinsed and washed with wash buffer 1 (0.1% BSA, 1 mM EDTA, pH 8, 40 mM sodium phosphate, pH 7.2, 5% SDS) for 30 min twice in a hybridization bottle in the hybridization oven, then washed buffer 2 (1 mM EDTA, pH 8; 40 mM sodium phosphate, pH 7.2; 1% SDS) twice for 30 min, wrapped in saran wrap and exposed to film. Endogenous mouse prion was utilized as a control.

### Western blotting

To prepare lysate, frozen gastrocnemius and spinal cord were homogenized in six volumes of the weight in homogenization buffer [50 mM Tris pH 7.5, 300 mM NaCl, 5 mM EDTA, 1% Triton, 1x protease inhibitor (Sigma, catalog #8340), 1x phosphatase inhibitor #1 (Sigma, catalog #P0044), 1x phosphatase inhibitor #2 (Sigma, catalog #P5726)] and stored in aliquots at − 80 °C to reduce freeze/thaw. An aliquot was further diluted to ten volumes of homogenization buffer. The samples were sonicated, and sodium dodecyl sulfate (SDS) was added to a final concentration of 2%. The samples were centrifuged for 20 min at 4 °C at 40,000 rpm. The supernatant was collected and protein concentration was determined using BCA protein assay (Pierce, Rockford, IL).

For MATR3 immunoblotting, 15 μg of protein from gastrocnemius or spinal cord were resolved on a 3–8% Tris-acetate gel and transferred onto a nitrocellulose membrane for 90 min at 200 mA. The blots were blocked in 5% milk in Tris-buffered saline (TBS) for an hour and were incubated overnight with rabbit α-MATR3 antibody (1:10,000, Novus Biologicals, catalog #NB100–1761) or mouse α-GAPDH antibody (1:5000, Meridian Life Sciences, catalog #H86504M) diluted in 5% milk/TBS. Following washes in TBS, blots were incubated with horseradish peroxidase-conjugated α-rabbit or α-mouse for one hour (1:5000, Jackson ImmunoResearch Laboratories Inc., α-rabbit catalog #711–036-125, α-mouse catalog #715–036-150). Membranes were then washed and incubated with ECL-Plus reagent (Fisher, catalog #509049326) and imaged on PXi using the GeneSys software (SynGene). When comparisons were performed across lines which had variable levels of degeneration, coomassie stain was used on 3–8% Tris-Acetate gels replicating the sample layout for gastrocnemius to confirm equivalent loading. When westerns were run within lines and age, immunoblotting for mouse α-GAPDH was utilized for a load control. Western analysis on spinal cord in the lead lines was run using GAPDH as the loading control.

### Immunohistochemistry

The formalin-fixed gastrocnemius and spinal cord were paraffin embedded and sectioned into 5 μm sections. Sections were deparaffinized and hydrated before antigen retrieval using either steaming in citrate buffer (10 mM citrate acid with 0.05% Tween-20, pH 6.0) for 30 min for MATR3 and GFAP or incubation in 10% formic acid for 10 min followed by a 20 min rinse under running water for Iba1. Following alternating washes in water and PBS, sections were blocked in 0.3% H_2_O_2_/PBS for 20 min. After thorough washes in Milli-Q H_2_O, sections were blocked with 10% normal goat serum (Vector Labs, catalog #S1000) in PBS and 0.05% Tween-20 (PBS-T) for 30 min at room temperature. Sections were then incubated with primary antibody [rabbit α-MATR3 to detect both human and murine MATR3, 1:1000, Novus Biologicals, catalog #NB100–1761; rabbit α-GFAP, 1:1000, Dako, catalog #Z0334; rabbit α-Iba-1, 1:500, Wako, catalog #019–19,741] in 5% normal goat serum/PBS-T overnight at 4 °C. Subsequently, the sections were rinsed with PBS-T and incubated with biotinylated α-rabbit antibody (1:500, Vector Labs, catalog #BA-1000) in 5% normal goat serum/PBS-T for 30 min at room temperature. The signal was detected with a standard peroxidase ABC system (Vector Labs, catalog #PK6100) with DAB as the chromogen, counterstained with hematoxylin, dehydrated and cover-slipped using Cytoseal (Thermo Scientific, catalog #8310–4).

### Quantification

For Northern blot, boxes were drawn around each *MATR3* and *PrP* mRNA band three times using Alpha View SA software (Protein Simple). Tg samples were then normalized by dividing the *MATR3* signal by *PrP* signal, then averaged between the same sample. For muscle Western blots, boxes of equal size were drawn around MATR3 bands using GeneTools software (SynGene) and corrected for background. For spinal cord western blots, boxes of equal size were drawn around MATR3 and, when utilized, GAPDH bands using GeneTools software (SynGene), corrected for background, and densitometric values were normalized by dividing the MATR3 signal by GAPDH. Expression level values for ~ 120 doublet, 90, 75, and 55 kDa were summed to yield total MATR3 levels.

**Fig. 1:**
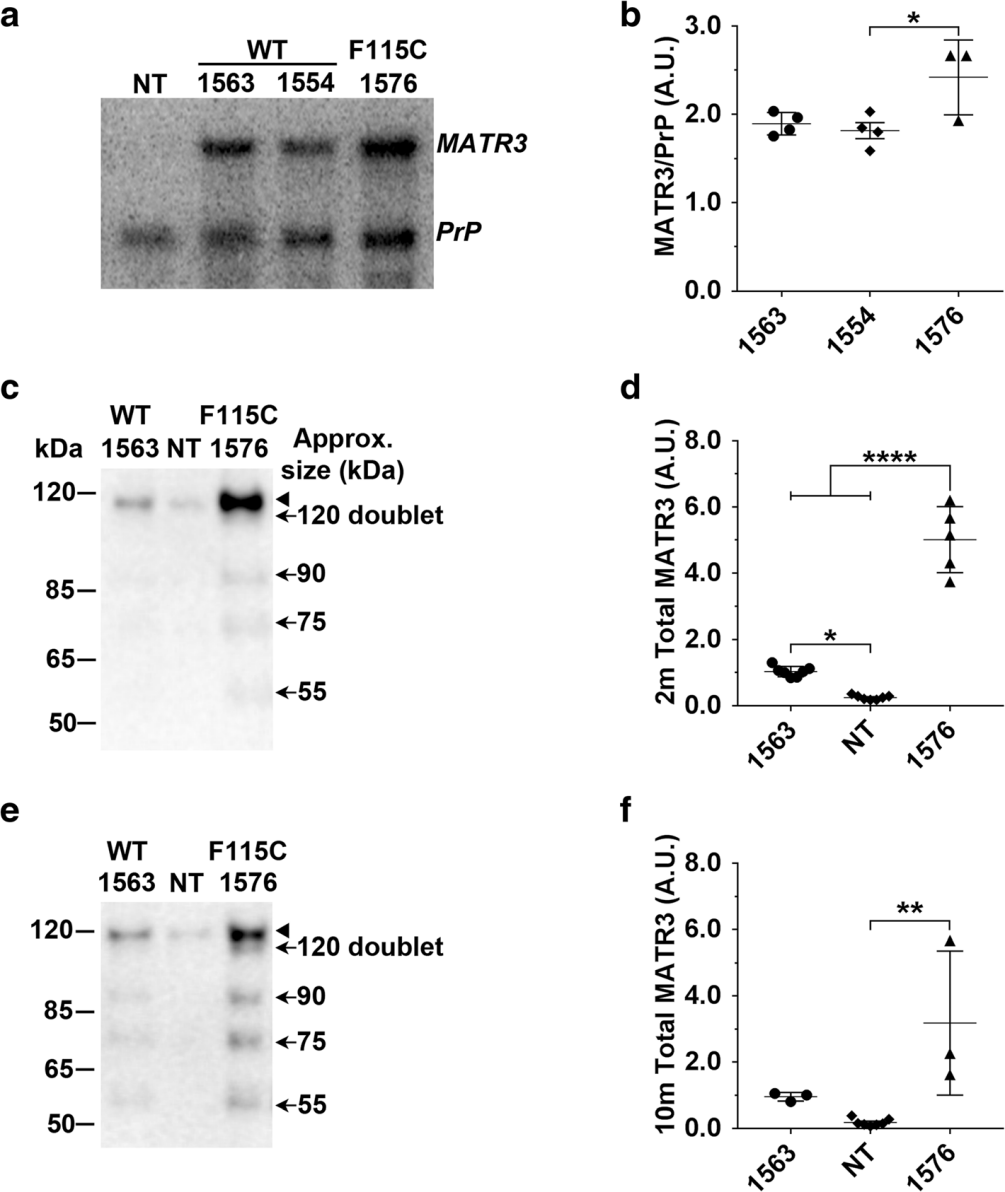
MATR3 is overexpressed in the muscle of MATR3^WT^ and MATR3^F115C^ transgenic mice. **a** Northern blot analysis of quadriceps showed transgenic *MATR3* RNA levels. Endogenous murine *PrP* mRNA, which is recognized by the same probe used to detect transgenic mRNA, was utilized as a loading control. **b** Quantification of Northern blots confirmed significant increases in MATR3 levels from lines 1563, 1554, and 1576. MATR3 values were normalized to PrP. One-way ANOVA, *p* = 0.0308. Bonferroni’s multiple comparison test showed that lines 1576 and 1554 were significantly different (*p* < 0.05), while there was no difference between lines 1576 to 1563 and 1554 to 1563. **c** Representative western blot of gastrocnemius using an antibody that recognizes both human and murine MATR3 showed an increase in total MATR3 (120 doublet, 90, 75, and 55 kDa combined) in ~ 2 month old transgenic mice from MATR3^WT^ lead line 1563 and MATR3^F115C^ lead line 1576 compared to NT mice. **d** Quantification of western blot confirmed significant increase in total MATR3 levels (120 doublet, 90, 75, and 55 kDa combined) from MATR3^WT^ and MATR3^F115C^ lines compared to NT. One-way ANOVA, *p* ≤ 0.0001. Bonferroni’s multiple comparison test showed that total MATR3 levels from lines 1563 (*p* ≤ 0.05) and 1576 (*p* ≤ 0.0001) were statistically significant from NT. MATR3 levels from lead line 1576 were significantly elevated compared to 1563 (*p* ≤ 0.0001). **e** Western blot analysis of gastrocnemius showed an increase in MATR3 levels in ~ 10 month old transgenic mice from MATR3^F115C^ lead line 1576 compared to NT. **f** Quantification of western blot confirming significant increase in total MATR3 levels (120 doublet, 90, 75, and 55 kDa combined) from MATR3^F115C^ mice compared to NT mice. One-way ANOVA, *p* ≤ 0.01. Bonferroni’s multiple comparison test showed that line 1576 (*p* ≤ 0.0001) was statistically significant from NT. There is no significant difference between lead line 1576 compared to lead line 1563, as well as lead line 1563 compared to NT (*p* > 0.05). *, *p* ≤ 0.05; **, *p* ≤ 0.01, ****, *p* ≤ 0.0001. Arrow head indicates expected MATR3 band at 120 kDa, arrows indicate lower molecular weight species as indicated

### Statistical analysis

GraphPad Prism 5 and GraphPad Prism 6 software were used for statistical analysis and graphing. One-way ANOVAs, two-way ANOVAs, or t-tests were used as indicated to compare NT, MATR3^WT^, and MATR3^F115C^ values. *p* < 0.05 was established as the threshold for statistically significance. Bonferroni’s multiple comparison test was used. Error bars in figures show standard deviation.

## Results

We generated Tg mouse lines using a cDNA encoding either WT, F115C mutant, or S85C mutant MATR3 (MATR3^WT^, MATR3^F115C^, and MATR3^S85C^ respectively) under the control of the MoPrP.Xho vector. Six Tg founders for MATR3^WT^, five Tg founders for MATR3^F115C^, and five Tg founders for MATR3^S85C^ transmitted the transgene (Table 1); however, each line was mosaic with low penetrance and required substantial breeding to establish consistent lines. To determine which lines of mice expressed the transgene, we initially relied on immunoblotting of spinal cord and muscle tissues. We quickly observed that a subset of mice transgenic for MATR3^WT^ and MATR3^F115C^ had elevated levels of MATR3 in muscle compared to NT mice and then used immunoblotting of muscle to screen mice towards establishing stable lines that would be more thoroughly characterized (Table 1). In mice transgenic for MATR3^S85C^, we could not identify mice with robust levels of MATR3 in muscle or spinal cord compared to NT mice and ultimately discontinued breeding all mice harboring the MATR3^S85C^ transgene. Additionally, breeding patterns suggested that one of the MATR3^F115C^ lines (line 72) was likely integrated on the X-chromosome; therefore, the line was excluded from the study.

**Table 1:** Founder lines of MATR3^WT^ and MATR3^F115C^ mice used in the study

Transgene	Founder	Transmitting	Status	Use in paper	Reason for exclusion
MATR3^WT^	8	Yes	Extinct	None	No/low expression
	9	Yes	Extinct	None	No/low expression
	59	Yes	Back-up line	*Validation*	
	60	Yes	Extinct	None	No/low expression
	66	No	Extinct	None	Not transmitting
	1554	Yes	Extinct	*Validation*	
	**1563**	**Yes**	**Lead Line**	**Primary data**	
MATR3^F115C^	70	Yes	Extinct	None	No/low expression
	72	Yes	Extinct	None	X-Linked
	1573	Yes	Extinct	*Validation*	
	**1576**	**Yes**	**Lead Line**	**Primary data**	
	1579	Yes	Extinct	*Validation*	
MATR3^S85C^	209	Yes	Extinct	None	No/low expression
	202	Yes	Extinct	None	No/low expression
	203	Yes	Extinct	None	No/low expression
	257	Yes	Extinct	None	No/low expression
	275	Yes	Extinct	None	No/low expression

Bold indicates lead lines

In the MATR3^WT^ and MATR3^F115C^ lines we established, *MATR3* Tg mRNA was robustly expressed in the skeletal muscle (Fig. 1a). There was no statistically significant difference in human *MATR3* RNA levels between the lead MATR3^F115C^ line (1576) compared to the lead MATR3^WT^ line (1563) (Fig. 1a, b); however, *MATR3* Tg mRNA levels in the lead MATR3^F115C^ line (1576) were modestly higher than the levels in one of the MATR3^WT^ validation lines, 1554 (Fig. 1a, b; *p* < 0.05).

Total MATR3 protein levels in the skeletal muscle of ~ 2-month old mice from MATR3^WT^ lead line 1563 (*p* < 0.05) and MATR3^F115C^ (lead line 1576, *p* < 0.0001) were significantly elevated over that of NT mice (Fig. 1c-d). Compared to NT mice, MATR3 levels were increased ~ 4-fold (*p* < 0.05) in MATR3^WT^ mice and ~ 20-fold (*p* < 0.0001) in MATR3^F115C^ mice (Fig. 1c-d). At 10 months of age, total MATR3 protein levels were roughly 17-fold higher in MATR3^F115C^ (lead line 1576) than NT mice (Fig. 1e, f; *p* < 0.01). Across all MATR3 Tg lines, MATR3^F115C^ mice from line 1579 had the highest elevation of MATR3 protein levels at ~ 2 months of age compared to NT mice (Additional file 2: Figure S1a); however, line 1579 was only used for validation studies as they became moribund before breeding was possible.

**Fig. 2:**
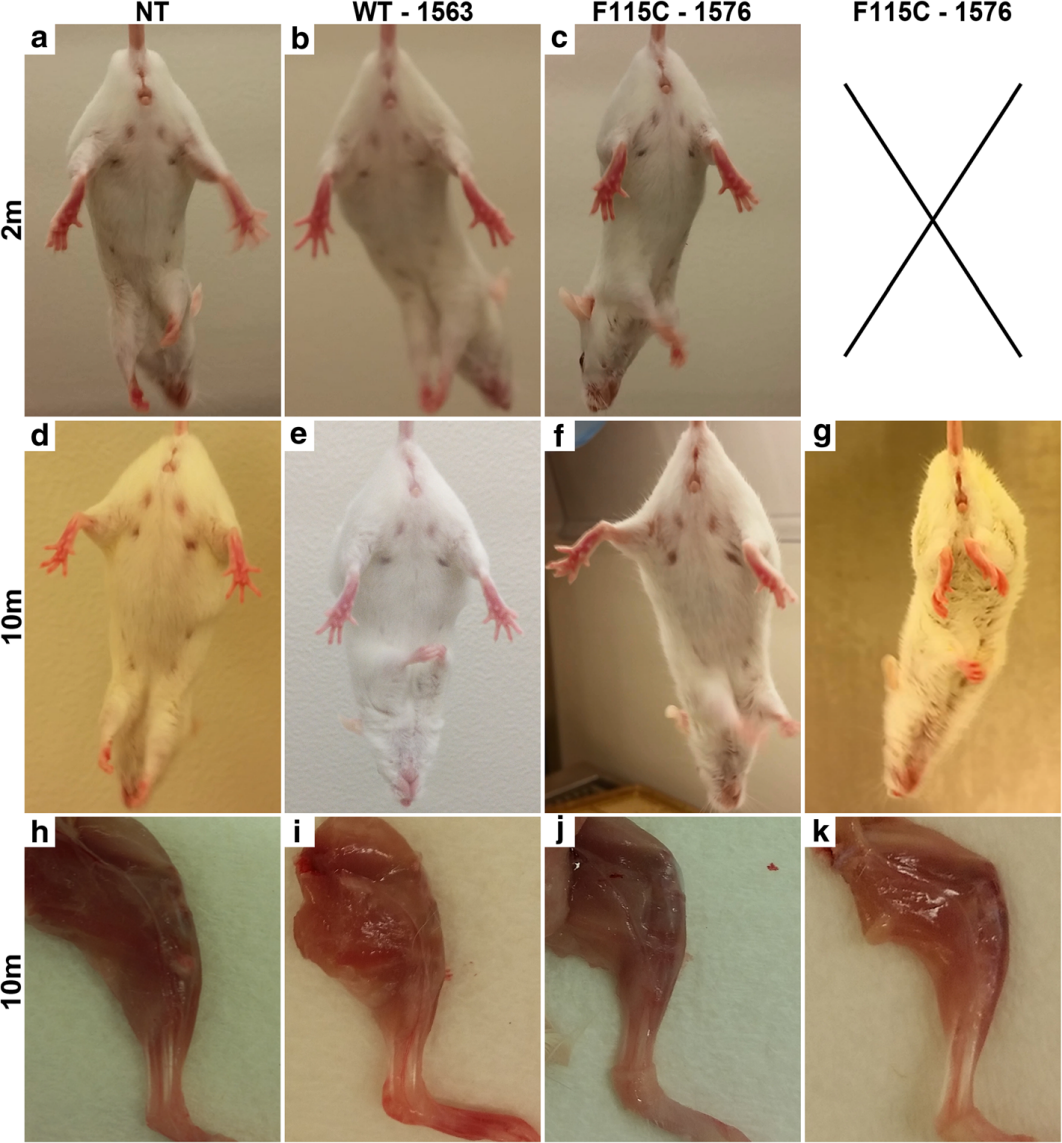
Severe phenotypic MATR3^F115C^ transgenic mice develop profound motor phenotype and show gross muscle atrophy. Escape extension of mice at ~ 2 months showed no differences comparing **a** NT to **b** MATR3^WT^ and **c** MATR3^F115C^ (lines noted on figure). Escape extension of mice at ~ 10 months showed no difference comparing **d** NT to **e** MATR3^WT^ displaying no obvious motor phenotype, and **f** MATR3^F115C^ displaying a mild-to-moderate phenotype. A subset of ~ 10-month old **g** MATR3^F115C^ mice lacked the normal escape reflex. Gross hindlimb muscle showed no difference between ~ 10 month old **h** NT and **i** MATR3^WT^; however, muscles appeared grossly atrophied in **j** MATR3^F115C^ displaying either a mild-to-moderate or **k** severe motor phenotype. Panels a-k are from female mice

During handling and while observing general cage behavior, Tg mice from MATR3^WT^ and MATR3^F115C^ lead lines initially were indistinguishable from NT littermates. At ~ 2 months of age, the escape extension in Tg mice from founder lines MATR3^WT^ (1563) and MATR3^F115C^ (1576) was generally comparable to NT mice (Fig. 2a-c). At ~ 10 months of age, the escape extension reflex in mice from MATR3^WT^ lead line 1563 was visually equivalent to that of NT controls (Fig. 2d, e). By ~ 10 months of age, mice from MATR3^F115C^ lead line 1576 typically presented with either no changes to escape extension (Fig. 2f) with observable mild-moderate impairment in walking behavior in home cage (not shown), or a severe change in escape extension (Fig. 2g) paired with striking difficulty while walking in the home cage (not shown). As early as ~ 4 months of age, a subset of MATR3^F115C^ mice from lead line 1576 appeared smaller in body size, with muscles in the hind limbs appearing smaller. Furthermore, they presented with difficulty gripping the edge of the cage. Similar motor impairment was observed in a subset of transgenic MATR3^F115C^ mice from validation lines 1573 (not shown) and 1579, which developed impairments as early as 0.9 months of age (Additional file 2: Figure S1c). In these young MATR3^F115C^ mice from validation line 1579, motor impairment rapidly progressed to hindlimb paresis and paralysis.

The body weight of MATR3^WT^ (lead line 1563) mice at ~ 2 and ~ 10 months of age was comparable to sex and age-matched NT controls (Additional file 3: Table S2). In contrast, body weights were reduced in MATR3^F115C^ mice from lead line 1576 compared to sex- and age-matched NT controls at both ~ 2 and ~ 10 months of age (Additional file 3: Table S2). Similarly, transgenic mice from MATR3^F115C^ validation line 1579 had reduced body weights compared to age-matched NT mice, although measurements were only compared for phenotypic males, given the young age of disease-onset (Additional file 3: Table S2).

**Fig. 3:**
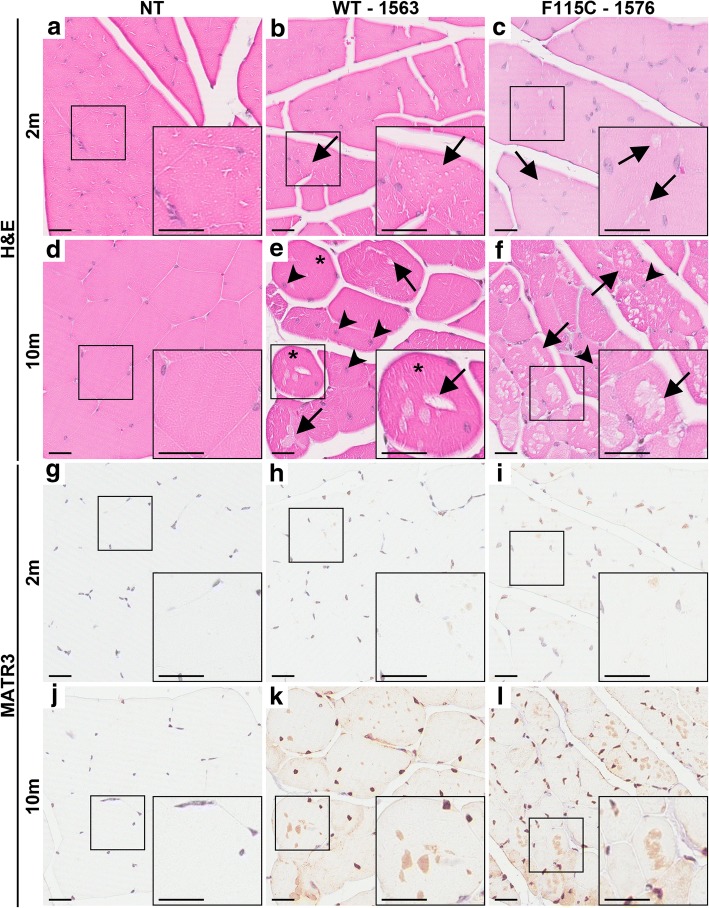
Muscle pathology is striking in ~ 10 month old MATR3^WT^ (lead line 1563) and MATR3^F115C^ (lead line 1576) transgenic mice. H&E of gastrocnemius at ~ 2 months of age from **a** NT, **b** MATR3^WT^, and **c** MATR3^F115C^ mice (lines noted on figure). Both the MATR3^WT^ and MATR3^F115C^ mice showed modest, early vacuolation in the fibers (arrow). H&E of the gastrocnemius at ~ 10 months of age from **d** NT, **e** MATR3^WT^ and **f** MATR3^F115C^ mice. Both MATR3^WT^ and MATR3^F115C^ mice showed striking pathology including vacuoles (arrow), internalized nuclei (arrow head), and rounded fibers (asterisks). MATR3 immunohistochemistry of the gastrocnemius at ~ 2 months of age from **g** NT, **h** MATR3^WT^, and **i** MATR3^F115C^. Both MATR3^WT^ and MATR3^F115C^ have increased immunostaining of MATR3 in the nucleus. MATR3 immunohistochemistry of the gastrocnemius ~ 10 months of age showed that compared to **j** NT mice, **k** MATR3^WT^ and **l** MATR3^F115C^ mice showed much more intense immunostaining of the nucleus, as well as robust cytoplasmic staining of MATR3. Scale bar measures 25 μm

Although the gastrocnemii of 10-month-old NT and MATR3^WT^ mice from lead line 1563 appeared grossly similar (Fig. 2h and i, respectively), gross muscle atrophy was apparent in ~ 10-month-old mice MATR3^F115C^ from lead line 1576. MATR3^F115C^ mice displaying the mild-to-moderate phenotype (Fig. 2j) showed moderate muscle atrophy, which was more pronounced in mice with the severe phenotype (Fig. 2k). MATR3^F115C^ mice from validation line 1579 also developed pronounced muscle atrophy (Additional file 2: Figure S1e) compared to NT mice (Additional file 2: Figure S1d). Transgenic MATR3^F115C^ mice (validation line 1579) which had severe motor abnormalities also showed severe myopathic changes (Additional file 2: Figure S1g) including rounded fibers, smaller fibers, centralized nuclei (white arrow head), and prominent vacuoles (white arrows) when compared to NT mice (Additional file 2: Figure S1f). To determine if myopathic changes were only present in mice with motor phenotypes, we examined muscle from MATR3^WT^ lead line 1563 and MATR3^F115C^ mice from lead line 1576 at ~ 2 and ~ 10 months of age (Fig. 3). The gastrocnemius of ~ 2-month-old MATR3^WT^ (lead line 1563) and MATR3^F115C^ (lead line 1576) showed vacuoles in the fibers compared to NT mice (Fig. 3a-c, black arrows) which appeared to worsen with age (Fig. 3d-f) based on the increased presence of rounded fibers (asterisks), internalized nuclei (black arrowheads), and larger and abundant vacuoles in the fibers when compared to NT gastrocnemii. The pathology was qualitatively more severe in MATR3^F115C^ compared to MATR3^WT^ mice, especially at ~ 10 months of age.

Next, we sought to determine whether immunohistochemical techniques would also show increased MATR3 levels within the gastrocnemii of Tg MATR3 mice compared to NT mice, as indicated in earlier immunoblotting data. NT mice could be easily distinguished from MATR3^WT^ (lead line 1563) and MATR3^F115C^ (lead line 1576 and validation line 1579) mice based on MATR3 immunostaining of the muscle (Fig. 3g-l, Additional file 2: Figure S1h, i). In the lead MATR3^F115C^ line (1576) and MATR3^WT^ line (1563) at ~ 2 months of age, we observed MATR3 immunoreactivity heterogeneously in the nuclei of the gastrocnemius muscle fibers (Fig. 3h, i); however, in the muscle fibers of MATR3^WT^ and MATR3^F115C^ mice at ~ 10 months of age, there was extensive MATR3 immunostaining in the majority of the nuclei compared to NT mice (Fig. 3j-l). There was occasional MATR3 diffuse immunostaining to the cytoplasm in the ~ 2-month-old lead MATR3^WT^ and MATR3^F115C^ lines (not shown), but cytoplasmic MATR3 staining was robust and frequent in the ~ 10-month Tg muscles, seeming to reside within the vacuoles (Fig. 3k, l).

**Fig. 4:**
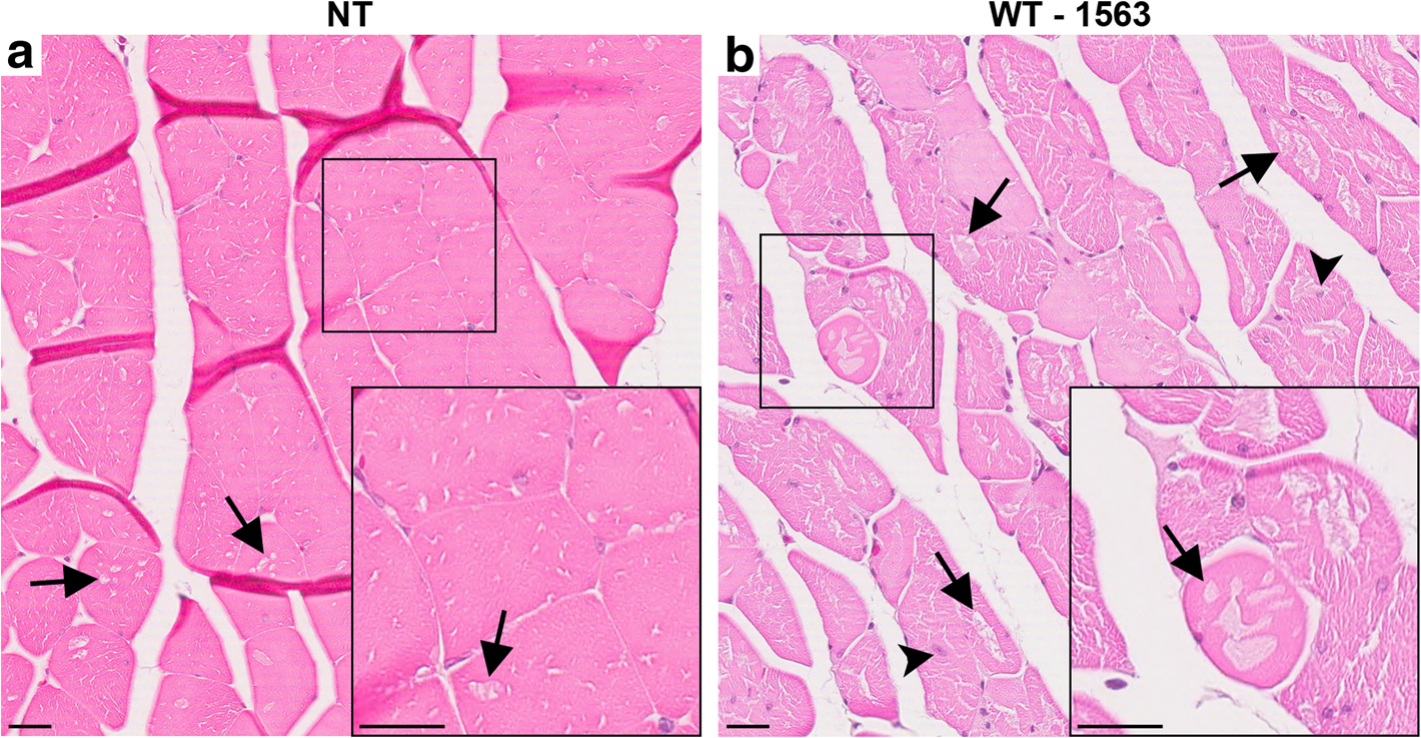
Striking muscle pathology in aged MATR3^WT^ (lead line 1563) mice. H&E stain of gastrocnemii of **a** NT at 24.6 months and **b** MATR3^WT^ from lead line 1563 at 22.7 months. While the NT muscle fibers showed some vacuoles (arrows), the MATR3^WT^ muscle showed striking pathology with abundant subsarcolemmal vacuoles, internalized nuclei (arrow heads), and loss of fiber shape. Scale bar measures 25 μm

Although the MATR3^F115C^ mice exhibited abbreviated life spans due to motor impairment, the MATR3^WT^ mice live through 20 months of age without obvious motor abnormalities. To determine whether the muscle pathology in MATR3^WT^ mice would eventually progress with age to the severity of that observed in the MATR3^F115C^ mice, we performed H&E staining on gastrocnemii of MATR3^WT^ mice from lead line 1563 at > 20 months of age (Fig. 4). Age matched NT muscle fibers showed occasional vacuolation consistent with aging (Fig. 4a, black arrows), but most of the 20-month-old MATR3^WT^ mice examined showed extensive muscle pathology including internalized nuclei (black arrowheads) as well as rounded and vacuolated fibers that, in some cases, appeared almost destroyed (Fig. 4b). Surprisingly, these myopathic features were observed in the MATR3^WT^ mice in the absence of obvious motor abnormalities.

In all MATR3 lines, we utilized the MoPrP to drive expression of the transgene as it has reproducibly been shown to drive transgene overexpression in the central nervous system [3, 11, 24, 25] with lower levels in the skeletal muscle [3]. Unexpectedly, MoPrP-driven MATR3 overexpression in the current study appeared to be minimal in the spinal cord of Tg mice from all lines examined. Northern blots showed minimal expression of the *MATR3* Tg mRNA in spinal cord from MATR3^WT^ (lead line 1563 and validation line 1554) and MATR3^F115C^ (lead line 1576), using endogenous *PrP* mRNA as a comparison (Additional file 4: Figure S2a). RNA was not available from MATR3^F115C^ validation line 1579; however, immunoblotting indicated no significant change in total MATR3 protein levels in the spinal cords of ~ 1.2-month-old Tg mice compared to NT mice (Additional file 4: Figure S2b, c).

Although northern and immuno- blot analyses did not reveal an overt increase in MATR3 levels in spinal cords of Tg MATR3 mice, we utilized immunohistochemistry to determine whether the MATR3 protein localization and glial profiles may be altered in the spinal cord of Tg mice compared to NT mice. Using an antibody that recognizes both murine and human MATR3, immunoreactivity was generally similar between Tg and NT mice, primarily localizing to spinal cord nuclei in NT, MATR3^WT^ (lead line 1563), and MATR3^F115C^ (lead line 1576) mice (Additional file 5: Figure S3a-c). Glial profiles in the spinal cords of Tg MATR3 lines were largely unremarkable compared to NT spinal cord (Additional file 5: Figure S3d-i).

## Discussion

We characterized three transmitting lines of Tg mice expressing full-length WT human MATR3 (MATR3^WT^) and three transmitting lines of Tg mice expressing full-length mutant (F115C) human MATR3 (MATR3^F115C^). All of our Tg founder lines were mosaic and required substantial outcrossing to develop consistent lead lines for MATR3^WT^ (lead line 1563) and MATR3^F115C^ (lead line 1576), both of which have robustly elevated levels of total MATR3 protein in their skeletal muscle, but not spinal cords. Both MATR3^WT^ and MATR3^F115C^ mice showed initial muscle pathology by 2 months of age that noticeably progressed by 10 months of age to include frequent, large vacuoles, rounded fibers, and internalized nuclei. Muscle pathology in the MATR3^WT^ mice appeared to lag behind MATR3^F115C^ mice, requiring additional aging to > 20 months of age before approximating the severity observed in the younger MATR3^F115C^ counterparts. Only MATR3^F115C^ mice develop a consistent, overt motor phenotype, which included paresis and paralysis. Motoric changes were accompanied by gross muscle atrophy, which could serve as one explanation for the body weight differences between NT mice and MATR3^F115C^ mice and was not observed in the MATR3^WT^ mice. Since MATR3^WT^ and MATR3^F115C^ lacked robust elevation or relocalization of MATR3 in the spinal cord and the overall glial profiles did not differ from NT mice, it appears that the transgenic MATR3 expression the in the muscle most likely led to myopathy rather than a primary motor neuron disease.

Although the human *MATR3* RNA expression levels in the skeletal muscle were not significantly different between the MATR3^F115C^ lead line (1576) and MATR3^WT^ lead line (1563), MATR3^F115C^ mice (lead line 1576) have a ~ 4-fold elevation in total MATR3 protein levels over MATR3^WT^ mice (lead line 1563) at ~ 2 months of age. Since the MATR3 antibodies that are commercially available detect both human and murine MATR3, it is impossible to know if the higher level of MATR3 protein in the mutant mice arise from induction of endogenous MATR3 expression or higher levels of human transgenic F115C MATR3 or an elevation of both murine and the transgenic MATR3. The elevation in the levels of MATR3 protein in the muscle MATR3^F115C^ mice (lead line 1576) over MATR3^WT^ mice (lead line 1563) was not proportional to the Tg mRNA levels between the two lead lines. This may suggest a disparity between translational regulation of WT versus F115C MATR3. Alternatively, it is possible that MATR3^F115C^ may turnover more slowly than MATR3^WT^, resulting in high steady-state levels of protein. Finally, it is possible that there is autoregulation of MATR3 expression, at some unknown level, in these lines and that WT and mutant MATR3 differ in their capacity to perform this function.

In immunoblots of skeletal muscle from younger NT and MATR3 Tg mice, we observed multiple MATR3-immunopositive bands (~ 120 doublet, 90, 75, 55 kDa). The origin of these bands is presently unclear. Since Tg MATR3 was expressed from a cDNA construct, the Tg *MATR3* RNA should not splice to produce alternative transcripts, and no such transcripts were observed on Northern blots. The ~ 120 kDa MATR3-immunopositive band is found in NT muscle as well as variety of other NT tissues [15]. Given these observations, the most likely origins of the lower MATR3-immunopositive species could be from either post-translational modification/proteolysis of the full-length protein or from alternative splicing of endogenous murine MATR3 that was induced by some means.

A surprising finding in these transgenic mice is that MATR3 was consistently elevated in the muscle, but not the spinal cord, despite the use of the MoPrP to drive transgene expression. The MoPrP-driven transgene overexpression is typically focused in the spinal cord and brain [3, 11, 24, 25] with lower levels in the skeletal muscle [3]. This is in direct opposition to our current results, which could indicate that muscle and spinal cord may have different abilities to regulate overexpression or different levels of tolerance for MATR3 expression. It is possible that we missed transgenic founders having high levels of MATR3 in the spinal cord because they did not survive until birth.

Given that the levels of MATR3 were consistently elevated only in muscle of both MATR3^WT^ and MATR3^F115C^ mice, it is not surprising that the pathology within mice is primarily confined to the muscle. We observed extensive structural changes within the muscle of multiple lines of MATR3^WT^ and MATR3^F115C^ mice including pronounced vacuolation of the muscle fibers, internalized nuclei, rounded and ring fibers, variation in muscle fiber size, and gross muscle atrophy. Each of these changes is generally associated with a form of muscular dystrophy rather than being neurogenic in origin [8]. The muscle pathology, gross muscle atrophy and motor phenotype that was present in our MATR3^F115C^ Tg mice resembles that observed in the humans with VCPDM linked to mutant MATR3 [2, 5, 12, 13, 19, 26]. Interestingly, VCPDM has only been linked to the S85C mutation in MATR3 [2, 5, 12, 13, 19, 26]; whereas, the F115C mutation in MATR3 utilized in these transgenic mice has been linked to ALS. Muscle pathology in humans with S85C-linked VCPDM includes the presence of subsarcolemmal vacuoles, variable muscle fiber size, and an increase in internalize nuclei in the gastrocnemius or tibialis anterior [2, 5, 26]. We observed these features in muscle of MATR3^F115C^ mice, and to a lesser degree in MATR3^WT^ mice. The ability of ALS-linked F115C MATR3 to cause overlapping pathologies and phenotypes to human VCPDM indicates that humans bearing any *MATR3* mutation should be examined for myopathy.

One limitation of these Tg mice is that there are no reported cases of myopathy or ALS in humans caused by an overexpression of MATR3. Thus, phenotypes and pathology driven by the overexpression of MATR3 in mice may be caused by different mechanisms than diseases found in humans with MATR3 mutations. We note that heterozygous MATR3 gene-trap mice have not been reported to show ALS- or VCPDM-like phenotypes [14]. In these gene trap mice, the longer transcript of *Matr3* was disrupted; however, a shorter *Matr3* transcript, which is found in the developing heart, was produced [14]. Since the gene-trap mice may not accurately represent a complete MATR3 knockout model, additional attempts for knockout or knock-in models should be pursued in order to determine if the ALS or VCPDM mutations in *MATR3* could result from a loss of function of the protein. Regardless, we do see a similar pathology in muscles of MATR3^WT^ and MATR3^F115C^ mice compared to the muscle pathology in the VCPDM patients, indicating that MATR3 overexpression certainly affected muscle fiber biology. Another limitation to these Tg mice concerns muscle development by overexpressing human MATR3. RT-PCR of heart has shown that levels of *MATR3* during development decreases by embryonic day 16.5 through postnatal day 0, then remain consistent through adulthood [14]. It is possible that by overexpressing human MATR3 early in development, the normal developmental processing of MATR3 is disturbed, and the muscle pathology and phenotype we observe could be a case of inappropriate development.

It is not unusual for the first or even second-generation murine models to only partially mimic aspects of neurodegenerative diseases, even in the context of pathologies that may not initially seem disease-relevant. For example, many of the initial TDP-43 Tg models helped elucidate multiple biologically-relevant features of TDP-43 such as its role in mitochondrial biology [24], despite serving as incomplete models for the human condition. Similarly, homozygous progranulin knockout mice develop neuronal ceroid lipofuscinosis [1]; however, a link between progranulin deficiency and human neuronal ceroid lipofuscinosis was not discovered until two years after we reported the link in the mouse model [20]. We anticipate that the current MATR3 models could provide similar insights.

Our data suggest that MATR3-linked diseases may lie on a spectrum between ALS and distal myopathy, and this spectrum is not without precedent. Valosin-containing protein (*VCP*) is another gene that lies on this spectrum of ALS to myopathy. Mutations in *VCP* cause inclusion body myopathy associated with Paget disease of bone and frontotemporal dementia (IBMPFD) [9]. Individuals with *VCP* mutations present with proximal and distal muscle paresis, similar to limb girdle muscular dystrophy, and muscle biopsy showing myopathic features included a change in muscle fiber size and rimmed vacuoles [9]; however, mutations in *VCP* can also lead to ALS, including two of the same mutations that had been previously linked to IBMPFD [6, 23].

## Conclusions

In aggregate, our studies provide the first Tg mouse models expressing any form of human MATR3 and demonstrate that simple dysregulation of MATR3 can cause robust structural abnormalities of the muscle. Our data suggest that MATR3 may have regulatory mechanisms that are influenced by tissue type and/or mutation status. These novel MATR3 models will provide critical tools to explore MATR3 regulation and its role in normal and disease biology. Given that the F115C mutation in MATR3 has not been linked to myopathy in humans, our data support the possibility that *MATR3* mutations could cause diseases that lie in a spectrum between ALS and myopathy and that any individual containing a *MATR3* mutation should be assessed for myopathic changes, regardless of clinical diagnosis.

## Additional files

Additional file 1: **Table S1.** Animals used in the study. *Indicates genotype has been confirmed by sequencing. ^§^ indicates genotype has been confirmed by restriction digest. Bold indicates when data from animal is pictured within figure. Geno., genotype; Gen., generation; Pheno., phenotype; NP, no phenotype; MM, mild-to-moderate phenotype; S, severe phenotype; F, female; M, male; mo, month; Fig, Figure; SF, Supplementary Figure; T, Table; ST, Supplementary Table. (DOCX 37 kb)

Additional file 2: **Figure S1.** Muscle immunobloting and pathology show striking differences between MATR3^F115C^ mice from validation line 1579 and NT mice. **a** Western blot analysis of gastrocnemius showed an increase in total MATR3 levels (~ 120 double, 90, 75, and 55 kDa combined) in mice ~ 1.5 months old transgenic mice from validation line 1579 compared to NT mice and mice from MATR3^F115C^ lead line 1576 and 1573. Escape extension showed differences between **b** NT compared to **c** MATR3^F115C^ transgenic mouse from validation line 1579 displaying a severe phenotype. Gross hindlimb muscle atrophy was apparent when comparing hindlimb of **d** NT to **e** MATR3^F115C^ mice. H&E of **f** NT and **g** MATR3^F115C^ where MATR3^F115C^ gastrocnemius showed striking pathology including centralized nuclei (white arrow head), rounded fibers, smaller fibers, and subsarcolemmal vacuoles (white arrows). Immunohistochemistry of **h** NT gastrocnemius and **i** MATR3^F115C^ showed that MATR3 immunoreactivity was elevated in the nucleus and cytoplasm of MATR3^F115C^ mice. Panels b-e are from males. Scale bar measures 25 μm. (TIF 4722 kb)

Additional file 3: **Table S2.** Average body weights for lead MATR3^WT^ and MATR3^F115C^ lines. MATR3^WT^ mice (lead line 1563) were not significantly different when compared to age-matched NT males (~ 2 mo.: two-way ANOVA, *p* > 0.05; ~ 10 mo.: t-test, p > 0.05) and females (~ 2 mo.: two-way ANOVA, *p* > 0.05). At ~ 2 months, MATR3^F115C^ mice (lead line 1576) weighed significantly less compared to age-matched and sex-matched NT mice (two-way ANOVA, *p* < 0.0001) and at ~ 10 months, male (two-way ANOVA, *p* < 0.0001) and female (two-way ANOVA, *p* < 0.05) MATR3^F115C^ mice (lead line 1576) weighed significantly less compared to age- and sex-matched NT mice. Not significant, n.s; *, *p* ≤ 0.05; ****, *p* ≤ 0.0001. (DOCX 16 kb)

Additional file 4: **Figure S2.** MATR3 is not robustly elevated in the spinal cords of MATR3^WT^ and MATR3^F115C^ mice. **a** Northern blot analysis of cervical spinal cord showed limited transgenic *MATR3* RNA expression in MATR3^WT^ (lead line 1563 and validation line 1554) and MATR3^F115C^ (lead line 1576) mice at ~ 4 months of age. Mouse prion (*PrP*) mRNA served as a loading control. **b** Western blot demonstrated that total MATR3 is not robustly elevated in spinal cord of MATR3^F115C^ (validation line 1579) mice compared to NT mice at ~ 1.5 months of age. GAPDH was utilized as a loading control. **c** Quantification of Western blot showed no significant difference in total MATR3 levels in spinal cord of NT compared to MATR3^F115C^ validation line 1579 mice (t-test, *p* > 0.05). (TIF 907 kb)

Additional file 5: **Figure S3.** Unremarkable spinal cord pathology in aged MATR3^WT^ and MATR3^F115C^ mice. MATR3 immunohistochemistry of spinal cord at ~ 10 months of age from **a** NT, **b** MATR3^WT^ from lead line 1563, and **c** MATR3^F115C^ from lead line 1576. Qualitatively, there appeared to be no difference in Iba-1 immunohistochemistry between **d** NT, **e** MATR3^WT^, and **f** MATR3^F115C^, or in GFAP immunohistochemistry of **g** NT, **h** MATR3^WT^, and **i** MATR3^F115C^ spinal cords. Scale bar measures 100 μm. (TIF 6303 kb)

## Abbreviations

Mo: promoter
PrP: mouse prion
ALS: Amyotrophic lateral sclerosis
*C9orf72*: Chromosome 9 open reading frame gene
CNS: Central nervous system
F: Female
F115C: Phe115Cys. ALS causing mutation in MATR3
fALS: Familial ALS
Fig: Figure
*FUS*: fused in sarcoma gene
GAPDH: Glyceraldehyde 3-phosphatase dehydrogenase
Gen.: Generation
Geno.: Genotype
GFAP: Glial fibrillary acidic protein
H&E: Hematoxylin and eosin stain
Iba-1: allograft inflammatory factor 1, or ionized calcium-binding adapter molecule 1
IBMPFD: Inclusion body myopathy associated with Paget disease of bone and frontotemporal dementia
M: Male
*MATR3*: MATR3 gene
MATR3: Matrin 3, MATR3 protein
MATR3^F115C^: Transgenic mice expressing F115C mutant human MATR3
MATR3^S85C^: Transgenic mice expressing S85C mutant human MATR3
MATR3^WT^: Transgenic mice expressing wild-type human MATR3
MM: Mild to moderate phenotype
mo.: Month
NLS: Nuclear localization sequence
NP: No phenotype
NT: Non-transgenic
PBS: Phosphate buffered saline
Pheno.: Phenotype
PrP: Mouse prion
S: Severe phenotype
S85C: Ser85Cys. ALS and VCPDM causing mutation in MATR3
sALS: Sporadic ALS
SDS: Sodium dodecyl sulfate
SF: Supplementary Figure
*SOD1*: Superoxide dismutase I gene
ST: Supplementary Table
T: Table
*TARDBP*: Tar-DNA binding protein gene
TBS: Tris-buffered saline
TDP-43: TAR DNA binding protein 43
Tg: Transgenic
UTR: Untranslated region
VCP: Valosin containing protein
*VCP*: Valosin containing protein gene
VCPDM: autosomal dominant distal myopathy with vocal cord and pharyngeal weakness
WT: Wild-type
