# Supplementary material for: Analysis of spinal and muscle pathology in transgenic mice overexpressing wild-type and ALS-linked mutant MATR3
Source: Acta Neuropathol Commun. 2018 Dec 19;6:137. doi: 10.1186/s40478-018-0631-0 (PMC6299607; doi:10.1186/s40478-018-0631-0)
Supplement: Supplementary file 1 — Table S1. Animals used in the study. *Indicates genotype has been confirmed by sequencing. § indicates genotype has been confirmed by restriction digest. Bold indicates when data from animal is pictured within figure. Geno., genotype; Gen., generation; Pheno., phenotype; NP, no phenotype; MM, mild-to-moderate phenotype; S, severe phenotype; F, female; M, male; mo, month; Fig, Figure; SF, Supplementary Figure; T, Table; ST, Supplementary Table. (DOCX 37 kb) [file 40478_2018_631_MOESM1_ESM.docx]

**Supplementary Table 1.** Animals used in the study.

| Fo. | Animal ID | | Sex | Geno. | Gen. | Age (mo). | Pheno. | Body Weight (g) | Use (Figure / Table) |
| --- | --- | --- | --- | --- | --- | --- | --- | --- | --- |
| 59 | MF21.3 | | F | WT* | F1 | 2.3 | NP | 17.6 | Fig: 3; SF3 |
|  | MF21.4 | | M | WT* | F1 | 2.3 | NP | 22.8 | Fig: 3; SF3 |
|  | MF21.5 | | M | WT* | F1 | 2.3 | NP | 20.8 | Fig: 3; SF3 |
|  | MF21.7 | | M | WT* | F1 | 2.3 | NP | 18.4 | Fig: 3; SF3 |
|  | MF21.8 | | M | WT* | F1 | 2.3 | NP | 20.5 | Fig: 3; SF3 |
|  | F102.7 | | M | WT* | F4 | 11.5 | NP | 38.4 | Fig: 1e,f; 2; 3; SF3 |
|  | F102.8 | | M | WT* | F4 | 11.5 | NP | 33.2 | Fig: 1e,f; 2; 3; SF3 |
|  | F103.6 | | M | WT* | F4 | 11.5 | NP | 32.2 | Fig: 1e,f; 2; 3; SF3 |
|  | F123.10 | | M | WT | F4 | 19.4 | NP | 35.1 | Fig: 2 |
| 1554 | MF8.4 | | M | WT* | F2 | 2.4 | NP | 28.9 | Fig: 3; SF3 |
|  | MF8.7 | | M | WT* | F2 | 2.4 | NP | 27.6 | Fig: 3; SF3 |
|  | MF8.8 | | M | WT* | F2 | 2.4 | NP | 27.6 | Fig: 3; SF3 |
|  | MF8.9 | | M | WT* | F2 | 2.4 | NP | 27.3 | Fig: 3; SF3 |
|  | F108.1 | | F | WT* | F4 | 3.0 | NP | 24.2 | Fig: **1**a,**b**; **SF2a** |
|  | F108.6 | | M | WT* | F4 | 3.0 | NP | 30.4 | Fig: **1a**,**b**; **SF2a** |
|  | F104.2 | | F | WT* | F4 | 3.3 | NP | 24.9 | Fig: **1**a,**b**; **SF2a** |
|  | F104.5 | | F | WT* | F4 | 3.3 | NP | 25.3 | Fig: **1**a,**b**; **SF2a** |
|  | F113.1 | | F | WT* | F4 | 9.9 | NP | 27.6 | Fig: 1e,f; 2; 3; SF3 |
|  | F113.8 | | M | WT* | F4 | 9.9 | NP | 41.9 | Fig: 1e,f; 2; 3; SF3 |
|  | F113.9 | | M | WT* | F4 | 9.9 | NP | 38.8 | Fig: 1e,f; 2; 3; SF3 |
|  | F84.2 | | F | WT | F3 | 20.9 | NP | 50.5 | Fig: 2; 4 |
|  | F89.5 | | M | WT | F3 | 24.0 | NP | 36.5 | Fig: 4 |
|  | F84.3 | | F | WT | F3 | 25.3 | NP | 56.0 | Fig: 4 |
|  | F84.1 | | F | WT | F3 | 25.4 | NP | 35.8 | Fig: 2; 4 |
| 1563 | MF26.1 | | F | WT* | F2 | 2.0 | NP | 22.5 | Fig: 3; SF3 / T: **ST2** |
|  | MF26.3 | | M | WT* | F2 | 2.0 | NP | 25.3 | Fig: 3**b**,h; SF3 / T: **ST2** |
|  | MF26.4 | | M | WT* | F2 | 2.0 | NP | 24.1 | Fig: 3b,**h**; SF3 / T: **ST2** |
|  | MF26.8 | | M | WT* | F2 | 2.0 | NP | 26.6 | Fig: 3; SF3 / T: **ST2** |
|  | MF5.2 | | F | WT* | F1 | 2.1 | NP | 21.6 | Fig: 3; SF3 / T: **ST2** |
|  | MF5.3 | | F | WT* | F1 | 2.1 | NP | 19.1 | Fig: 3; SF3 / T: **ST2** |
|  | MF5.6 | | F | WT* | F1 | 2.1 | NP | 20.5 | Fig: 3; SF3 / T: **ST2** |
|  | MF5.7 | | M | WT* | F1 | 2.1 | NP | 24.9 | Fig: 3; SF3 / T: **ST2** |
|  | MF5.9 | | M | WT* | F1 | 2.1 | NP | 26.7 | Fig: 3; SF3 / T: **ST2** |
|  | MF36.2 | | F | WT* | F2 | 2.3 | NP | 19.3 | Fig: 3; SF3 / T: **ST2** |
|  | MF36.3 | | F | WT* | F2 | 2.3 | NP | 22.0 | Fig: 3; SF3 / T: **ST2** |
|  | MF36.5 | | F | WT* | F2 | 2.3 | NP | 20.3 | Fig: 3; SF3 / T: **ST2** |
|  | MF36.7 | | M | WT* | F2 | 2.3 | NP | 26.1 | Fig: 3; SF3 / T: **ST2** |
|  | MF19.2 | | F | WT* | F2 | 2.3 | NP | 21.6 | Fig: 3; SF3 / T: **ST2** |
|  | MF19.4 | | M | WT* | F2 | 2.3 | NP | 27.7 | Fig: 3; SF3 / T: **ST2** |
|  | MF19.8 | | M | WT* | F2 | 2.3 | NP | 25.9 | Fig: 3; SF3 / T: **ST2** |
|  | F189.1 | | F | WT | F8 | 2.4 | NP | 25.0 | Fig: **1c**,**d**; 2 |
|  | F189.2 | | F | WT | F8 | 2.4 | NP | 19.5 | Fig: 2 |
|  | F189.3 | | M | WT | F8 | 2.4 | NP | 30.6 | Fig: **1**c,**d**; 2 |
|  | F189.5 | | M | WT | F8 | 2.4 | NP | 27.8 | Fig: **1**c,**d**; 2 |
|  | F138.3 | | F | WT* | F6 | 2.5 | NP |  | Fig: **2b** |
|  | F187.1 | | F | WT | F7 | 2.8 | NP |  | Fig: **1**c,**d**; 2 |
|  | F187.3 | | F | WT | F7 | 2.8 | NP | 23.3 | Fig: **1**c,**d**; 2 |
|  | F187.5 | | F | WT | F7 | 2.8 | NP | 23.2 | Fig: **1**c,**d**; 2 |
|  | F187.9 | | M | WT | F7 | 2.8 | NP | 30.5 | Fig: **1**c,**d**; 2 |
|  | F111.1 | | F | WT* | F4 | 3.9 | NP | 27.0 | Fig: **1**a,**b**; **SF2a** |
|  | F111.2 | | F | WT* | F4 | 3.9 | NP | 25.1 | Fig: **1**a,**b**; **SF2a** |
|  | F111.6 | | M | WT* | F4 | 4.7 | NP | 32.1 | Fig: **1a**,**b**; SF2a |
|  | F111.7 | | M | WT* | F4 | 4.7 | NP | 35.4 | Fig: **1**a,**b**; SF2a |
|  | F148.6 | | F | WT | F7 | 9.9 | NP |  | Fig: **2e** |
|  | F132.5 | | M | WT | F5 | 10.4 | NP | 39.2 | T: **ST2** |
|  | F105.6 | | M | WT* | F4 | 10.6 | NP | 46.5 | Fig: 2; 3; SF3 / T: **ST2** |
|  | F101.7 | | F | WT* | F4 | 10.7 | NP | 30.1 | Fig: **1e**,**f**; 2; 3e,k; SF3 |
|  | F101.9 | | M | WT* | F4 | 10.7 | NP | 33.8 | Fig: 1e,**f**; 2; 3; SF3 / T: **ST2** |
|  | F101.10 | | M | WT* | F4 | 10.7 | NP | 32.8 | Fig: 1e,**f**; 2; 3; SF3 / T: **ST2** |
|  | MF14.1 | | F | WT | F2 | 13.0 | NP | 57.6 | Fig: 2; 4 |
|  | F91.3 | | M | WT | F3 | 13.3 | NP | 43.8 | Fig: 4 |
|  | F132.5 | | M | WT | F5 | 15.8 | NP | 41.7 | Fig: 2 |
|  | MF13.11 | | M | WT | F1 | 15.8 | NP | 33.3 | Fig: 2 |
|  | F91.5 | | M | WT* | F3 | 17.7 | NP | 33.2 | Fig: 2; 4 |
|  | F118.9 | | M | WT^§^ | F4 | 16.9 | NP | 32.8 | Fig: 2 |
|  | F91.7 | | M | WT* | F3 | 17.7 | NP | 23.0 | Fig: 2; 4 |
|  | MF4.7 | | F | WT | F1 | 19.4 | NP | 27.7 | Fig: 2; 4 |
|  | MF3.6 | | M | WT | F1 | 19.7 | NP | 29.0 | Fig: 2; 4 |
|  | MF14.4 | | M | WT* | F2 | 20.0 | NP | 42.4 | Fig: 2; 4 |
|  | F95.6 | | M | WT* | F3 | 20.5 | NP | 34.3 | Fig: 2; 4 |
|  | MF13.1 | | F | WT | F1 | 21.0 | NP | 41.6 | Fig: 2; 4 |
|  | MF4.5 | | F | WT | F1 | 21.2 | NP | 22.2 | Fig: 2; 4 |
|  | F95.5 | | F | WT | F3 | 21.3 | NP | 25.9 | Fig: 2; 4 |
|  | F87.3 | | F | WT* | F3 | 21.5 | NP | 27.4 | Fig: 2; 4 |
|  | MF24.5 | | M | WT | F1 | 21.7 | NP | 34.0 | Fig: 2; 4 |
|  | F57.4 | | M | WT* | F2 | 22.1 | NP | 41.4 | Fig: 2; 4 |
|  | MF25.4 | | F | WT* | F2 | 22.4 | NP | 21.5 | Fig: 2; 4 |
|  | MF3.1 | | F | WT* | F1 | 22.7 | NP | 28.7 | Fig: 2; **4b** |
|  | MF13.8 | | F | WT | F1 | 27.3 | NP | 29.2 | Fig: 2; 4 |
|  | MF13.2 | | F | WT* | F1 | 27.6 | NP | 24.3 | Fig: 2; 4 |
|  | MF13.5 | | F | WT* | F1 | 29.2 | NP | 18.8 | Fig: 2; 4 |
|  | MF13.7 | | F | WT* | F1 | 33.1 | NP | 27.2 | Fig: 2; 4 |
| 1573 | MA24.3 | | F | F115C* | F2 | 2.2 | NP | 16.1 | Fig: 3; SF3 |
|  | MA24.9 | | M | F115C* | F2 | 2.2 | NP | 21.8 | Fig: 3; SF3 |
|  | MA24.4 | | M | F115C* | F2 | 2.6 | NP | 21.2 | Fig: 3; SF3 |
|  | MA95.3 | | F | F115C* | F3 | 7.3 | MM | 19.9 | Fig: 2; 3; SF3 |
|  | MA16.2 | | M | F115C* | F2 | 8.4 | S | 16.6 | Fig: 2; **SF1a** |
|  | MA2.7 | | M | F115C*^§^ | F1 | 9.2 | S | 20.1 | Fig: **1**e,**f**; 2; 3; **SF1a**; SF3 |
|  | MA95.5 | | M | F115C* | F3 | 9.7 | S | 17.2 | Fig: **1**e,**f**; 2 |
|  | MA71.7 | | M | F115C* | F3 | 10.2 | S | 19.0 | Fig: **1**e,**f**; 2 |
|  | MA16.1 | | F | F115C*^§^ | F2 | 13.8 | S | 16.9 | Fig: 2; 3; SF3 |
|  | MA78.5 | | M | F115C* | F3 | 18.5 | MM | 22.2 | Fig: 2 |
|  | MA95.4 | | M | F115C | F3 | 19.6 | S | 21.0 | Fig: 2 |
| 1576 | MA172.3 | | F | F115C | F9 | 1.8 | NP | 14.8 | T: **ST2** |
|  | MA172.6 | | F | F115C | F9 | 1.8 | NP | 17.2 | T: **ST2** |
|  | MA172.7 | | M | F115C | F9 | 1.8 | NP | 18.1 | T: **ST2** |
|  | MA172.8 | | M | F115C | F9 | 1.8 | NP | 20.1 | T: **ST2** |
|  | MA172.10 | | M | F115C | F9 | 1.8 | NP | 19.2 | T: **ST2** |
|  | MA171.2 | | F | F115C | F9 | 1.8 | NP | 15.8 | T: **ST2** |
|  | MA171.3 | | F | F115C | F9 | 1.8 | NP | 16.8 | T: **ST2** |
|  | MA171.4 | | F | F115C | F9 | 1.8 | NP | 18.0 | T: **ST2** |
|  | MA171.6 | | M | F115C | F9 | 1.8 | NP | 20.2 | T: **ST2** |
|  | MA184.3 | | F | F115C | F10 | 1.8 | NP | 14.8 | Fig: **1**c,**d**; 2 |
|  | MA184.4 | | M | F115C | F10 | 1.8 | NP | 18.5 | Fig: **1**c,**d**; 2 |
|  | MA27.1 | | F | F115C* | F2 | 1.8 | NP | 17.8 | Fig: **3c**,**i**; SF3 / T: **ST2** |
|  | MA27.5 | | M | F115C* | F2 | 1.8 | NP | 21.6 | Fig: 3; SF3 / T: **ST2** |
|  | MA27.7 | | M | F115C* | F2 | 1.8 | NP | 23.1 | Fig: 3; SF3 / T: **ST2** |
|  | MA182.1 | | F | F115C | F9 | 2.0 | NP | 16.3 | Fig: **1**c,**d**; 2 |
|  | MA146.5 | | M | F115C* | F7 | 3.1 | NP | 21.1 | Fig: **1a**,**b**; **SF2a** |
|  | MA146.6 | | M | F115C* | F7 | 3.1 | NP | 19.3 | Fig: 1a,b; **SF2a** |
|  | MA146.8 | | M | F115C* | F7 | 3.1 | NP | 18.2 | Fig: **1**a,**b**; **SF2a** |
|  | MA160.2 | | F | F115C* | F7 | 3.1 | NP |  | Fig: **2c** |
|  | MA181.2 |  | M | F115C | F10 | 3.1 | NP | 20.8 | Fig: **1**c,**d**; 2 |
|  | MA180.1 | | F | F115C | F9 | 3.6 | NP | 17.6 | Fig: **1c**,**d**; 2 |
|  | MA183.3 | | M | F115C | F10 | 3.6 | S | 11.2 | Fig: 2 |
|  | MA39.3 | | M | F115C* | F2 | 4.1 | S | 20.1 | Fig: 2; 3; **SF1a**, SF3 |
|  | 1576 | | F | F115C* | Founder | 4.8 | S | 30.1 | Fig: 2; 3; **SF1a**, SF3 |
|  | MA158.6 | | M | F115C* | F8 | 8.9 | S | 17.9 | Fig: 2 |
|  | MA165.3 | | F | F115C | F8 | 9.6 | S | 18.1 | Fig: 2 / T: **ST2** |
|  | MA158.4 | | M | F115C | F8 | 10.0 | S | 18.0 | Fig: 2 / T: **ST2** |
|  | MA160.2 | | F | F115C | F7 | 10.1 | MM | 14.8 | T: **ST2** |
|  | MA75.2 | | F | F115C* | F3 | 10.1 | MM | 27.3 | Fig: **1e**,**f**; 2; **3c**,**l**; SF3 / T: **ST2** |
|  | MA75.3 | | F | F115C* | F3 | 10.1 | MM | 20.1 | Fig: 2; 3; SF3 / T: **ST2** |
|  | MA75.4 | | F | F115C* | F3 | 10.1 | MM | 27.2 | Fig: **1**e,**f**; **2f**,**j**; 3; SF3 / T: **ST2** |
|  | MA158.5 | | M | F115C | F8 | 10.3 | S | 16.1 | T: **ST2** |
|  | MA39.1 | | F | F115C* | F2 | 10.5 | S | 17.7 | Fig: 2; 3; SF3 / T: **ST2** |
|  | MA128.1 | | F | F115C* | F6 | 10.6 | S | 18.7 | Fig: **2g**,**k** / T: **ST2** |
|  | MA146.7 | | M | F115C | F7 | 10.8 | MM | 16.8 | Fig: **1**e,**f**; 2 |
|  | MA158.3 | | M | F115C | F8 | 10.0 | MM | 10.0 | Fig: 2 / T: **ST2** |
|  | MA158.5 | | M | F115C | F8 | 10.3 | S | 10.3 | Fig: 2; 3 / T: **ST2** |
|  | MA108.1 | | F | F115C* | F4 | 15.6 | MM | 17.4 | Fig: 2 |
| 1579 | MA22.7 | | M | F115C* | F1 | 0.9 | S | 13.2 | Fig: SF1c,e,g,i; SF3 / T: **ST2** |
|  | MA83.1 | | F | F115C* | F1 | 1.1 | S | 10.8 | Fig: SF1c,e |
|  | MA13.8 | | M | F115C*^§^ | F1 | 1.3 | S | 32.8 | Fig: **SF1a**,c,e,g,i; **SF2b,c**; SF3 / T: **ST2** |
|  | MA35.8 | | M | F115C* | F1 | 1.3 | S | 15.0 | Fig: **SF1a**,c,e,g,i; **SF2b,c**; SF3 / T: **ST2** |
|  | MA1.4 | | M | F115C* | F1 | 1.4 | S | 13.5 | Fig: **SF1a**,**c**,e,g,i; SF3 / T: **ST2** |
|  | MA7.8 | | M | F115C*^§^ | F1 | 1.4 | S | 16.6 | Fig: **SF1a**,c,e,**g**,**i**; **SF2b**,**c**; SF3 / T: **ST2** |
| Control | MA1.5 | | M | NT |  | 1.4 |  | 25.2 | Fig: **SF1b**,d.f,h; SF3 / T: **ST2** |
|  | MA7.6 | | M | NT |  | 1.5 |  | 23.2 | Fig: **SF1a**b,d,f,h; **SF2b**,**c**; SF3 / T: **ST2** |
|  | MA82.7 | | M | NT |  | 1.5 |  | 24.4 | T: **ST2** |
|  | MA1.1 | | F | NT |  | 1.6 |  | 20.8 | Fig: **SF1a**,f,h; S3 |
|  | MA13.6 | | M | NT |  | 1.6 |  | 21.9 | Fig: **SF1a**,b,d,f,h; **SF2b**,**c**; S3 / T: **ST2** |
|  | MA172.1 | | F | NT |  | 1.8 |  | 21.5 | T: **ST2** |
|  | MA172.2 | | F | NT |  | 1.8 |  | 21.3 | T: **ST2** |
|  | MA172.4 | | F | NT |  | 1.8 |  | 20.9 | T: **ST2** |
|  | MA172.5 | | F | NT |  | 1.8 |  | 21.5 | T: **ST2** |
|  | MA172.9 | | M | NT |  | 1.8 |  | 25.0 | T: **ST2** |
|  | MA172.11 | | M | NT |  | 1.8 |  | 23.8 | T: **ST2** |
|  | MA171.1 | | F | NT |  | 1.8 |  | 21.1 | T: **ST2** |
|  | MA171.5 | | M | NT |  | 1.8 |  | 28.1 | T: **ST2** |
|  | F175.1 | | F | NT |  | 1.8 |  | 25.1 | T: **ST2** |
|  | F175.2 | | F | NT |  | 1.8 |  | 23.7 | T: **ST2** |
|  | F175.5 | | M | NT |  | 1.8 |  | 27.8 | T: **ST2** |
|  | MA184.1 | | F | NT |  | 1.8 |  | 20.4 | Fig: 1c,d; 2 |
|  | MA184.2 | | F | NT |  | 1.8 |  | 17.6 | Fig: 2 |
|  | MA184.6 | | M | NT |  | 1.8 |  | 21.5 | Fig: **1**c,**d**; 2 |
|  | MA12.1 | | F | NT |  | 1.9 |  | 21.2 | T: **ST2** |
|  | MA12.2 | | F | NT |  | 1.9 |  | 22.0 | T: **ST2** |
|  | MA12.4 | | F | NT |  | 1.9 |  | 20.3 | T: **ST2** |
|  | MA12.5 | | F | NT |  | 1.9 |  | 19.4 | T: **ST2** |
|  | MA35.7 | | M | NT |  | 2.0 |  | 26.0 | Fig: **SF1a**,b,d,f,h; **SF2b**,**c**; S3 / T: **ST2** |
|  | MF5.1 | | F | NT |  | 2.1 |  | 21.1 | Fig: 3; SF3 / T: **ST2** |
|  | MF5.8 | | M | NT |  | 2.1 |  | 28.5 | Fig: 3; SF3 / T: **ST2** |
|  | MA10.1 | | F | NT |  | 2.2 |  | 19.9 | T: **ST2** |
|  | MA10.7 | | M | NT |  | 2.2 |  | 26.7 | T: **ST2** |
|  | MA24.2 | | F | NT |  | 2.2 |  | 23.3 | Fig: **3**a,**g**; **SF1f**,**h**; S3 / T: **ST2** |
|  | F106.1 | | F | NT |  | 2.2 |  | 19.6 | Fig: 2; 3 / T: **ST2** |
|  | MA24.5 | | M | NT |  | 2.3 |  | 26.7 | Fig: 3 / T: **ST2** |
|  | MA24.6 | | M | NT |  | 2.3 |  | 26.1 | Fig: 3 / T: **ST2** |
|  | MA56.1 | | F | NT |  | 2.3 |  | 22.7 | T: **ST2** |
|  | MA56.6 | | M | NT |  | 2.3 |  | 25.0 | T: **ST2** |
|  | MA57.3 | | F | NT |  | 2.3 |  | 21.3 | T: **ST2** |
|  | MF21.1 | | F | NT |  | 2.3 |  | 18.7 | Fig: 3 |
|  | MF21.2 | | F | NT |  | 2.3 |  | 18.8 | Fig: 3 |
|  | MF21.6 | | M | NT |  | 2.3 |  | 22.1 | Fig: 3a,g |
|  | MA183.1 | | F | NT |  | 2.3 |  | 23.1 | Fig: **1**c,**d**; 2 |
|  | MA183.2 | | F | NT |  | 2.3 |  | 24.5 | Fig: **1**c,**d**; 2 |
|  | MF8.6 | | M | NT |  | 2.4 |  | 28.7 | Fig: 3 |
|  | F189.4 | | M | NT |  | 2.4 |  | 27.0 | Fig: **1**c,**d**; 2 |
|  | F187.2 | | F | NT |  | 2.8 |  | 23.2 | Fig: 2 |
|  | F187.4 | | F | NT |  | 2.8 |  | 22.2 | Fig: 2 |
|  | F187.6 | | M | NT |  | 2.8 |  | 27.7 | Fig: **1**c,**d**; 2 |
|  | F187.7 | | M | NT |  | 2.8 |  | 29.2 | Fig: 2 |
|  | F187.8 | | M | NT |  | 2.8 |  | 33.0 | Fig: 2 |
|  | F108.2 | | F | NT |  | 3.0 |  | 24.7 | Fig: **1**a,**b**; **S2a** |
|  | MA160.1 | | F | NT |  | 3.1 |  |  | Fig: **2a** |
|  | MA181.1 | | F | NT |  | 3.1 |  | 28.0 | Fig: **1c**,**d**; 2 |
|  | F111.5 | | M | NT |  | 4.7 |  | 33.1 | Fig: **1**a,**b** |
|  | F111.4 | | M | NT |  | 4.7 |  | 33.6 | Fig: SF2a |
|  | 1578 | | F | NT |  | 4.8 |  | 20.5 | Fig: 3; SF1a |
|  | F77.3 | | F | NT |  | 7.3 |  | 25.8 | Fig: 3 |
|  | MA16.4 | | M | NT |  | 8.6 |  | 39.4 | Fig: **SF1a** |
|  | F36.1 | | F | NT |  | 9.6 |  | 24.7 | Fig: 3; SF1a |
|  | F79.1 | | F | NT |  | 9.7 |  | 28.6 | Fig: 1e,**f**; **2d**,**h** |
|  | F113.10 | | M | NT |  | 9.9 |  | 40.6 | Fig: 1e,f; 2; 3 |
|  | F58.5 | | F | NT |  | 10.0 |  | 29.5 | Fig: 1e,f; 3 |
|  | F58.6 | | F | NT |  | 10.0 |  | 28.0 | Fig: **1**e,**f**; **3d**,**j** |
|  | MA75.1 | | F | NT |  | 10.1 |  | 31.6 | Fig: **1**e,**f**; 3 / T: **ST2** |
|  | F105.7 | | M | NT |  | 10.6 |  | 48.9 | Fig: 2; 3 / T: **ST2** |
|  | F105.8 | | M | NT |  | 10.6 |  | 40.9 | Fig: 2; 3 / T: **ST2** |
|  | F101.8 | | M | NT |  | 10.7 |  | 33.4 | Fig: **1e**,**f**; 2; 3 / T: **ST2** |
|  | F103.5 | | M | NT |  | 11.5 |  | 42.8 | Fig: 1e,f; 2; 3; S3 |
|  | F95.8 | | M | NT |  | 19.0 |  | 49.5 | Fig: 2; **4a** |
|  | F88.3 | | F | NT |  | 22.2 |  | 36.2 | Fig: 2; 4 |
|  | FVB | | M | NT |  | 24.6 |  | 36.0 | Fig: 2; 4 |

*Indicates genotype has been confirmed by sequencing. ^§^ indicates genotype has been confirmed by restriction digest. Bold indicates when data from animal is pictured within figure. Geno., genotype; Gen., generation; Pheno., phenotype; NP, no phenotype; MM, mild-to-moderate phenotype; S, severe phenotype; F, female; M, male; month, mo; Figure, Fig; Supplementary Figure, SF; Table, T; Supplementary Table, ST.
