# Supplementary figures and images for: Analysis of spinal and muscle pathology in transgenic mice overexpressing wild-type and ALS-linked mutant MATR3
Source: Acta Neuropathol Commun. 2018 Dec 19;6:137. doi: 10.1186/s40478-018-0631-0 (PMC6299607; doi:10.1186/s40478-018-0631-0)

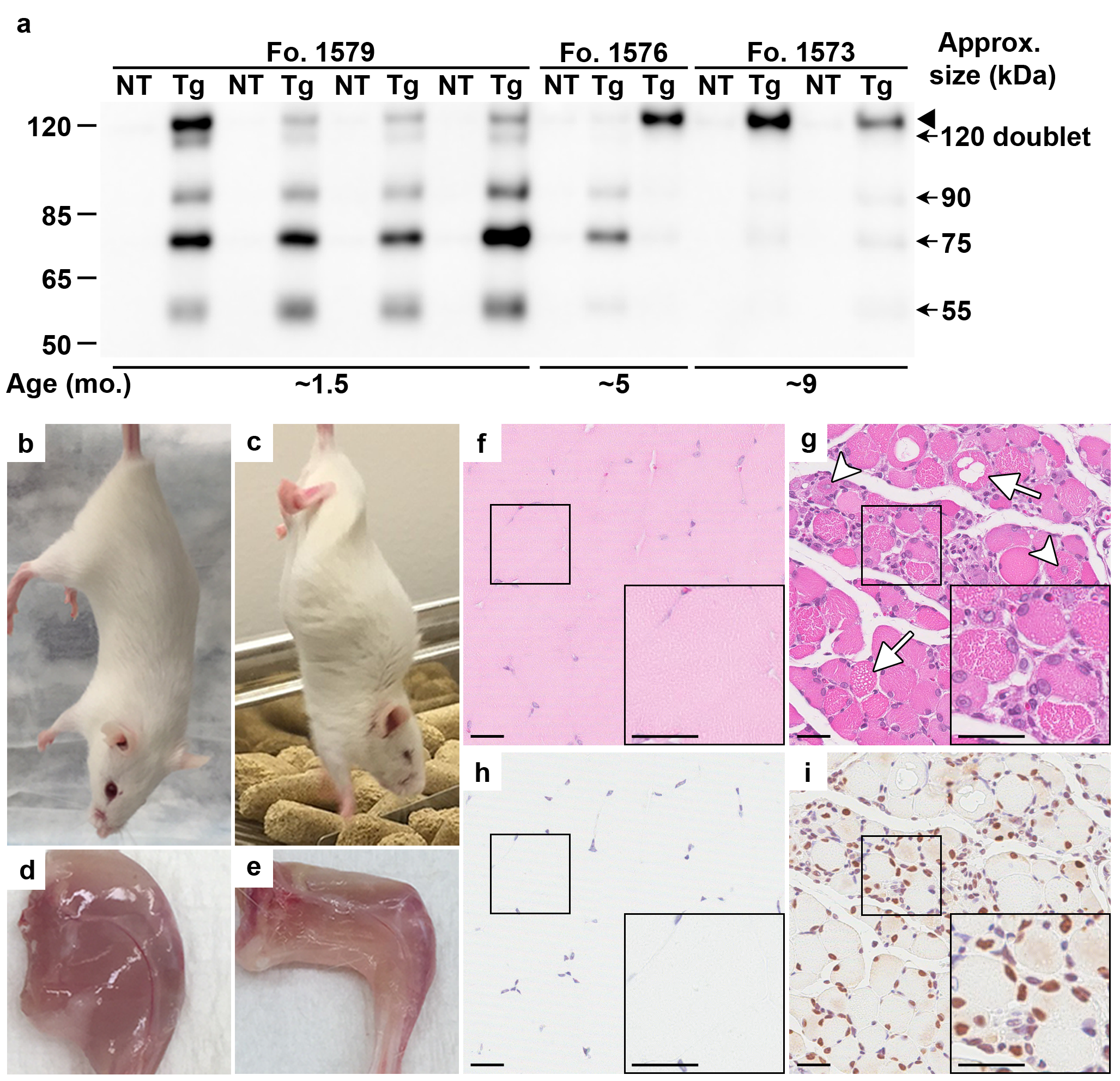

Supplement: Supplementary file 2 — Figure S1. Muscle immunobloting and pathology show striking differences between MATR3F115C mice from validation line 1579 and NT mice. a Western blot analysis of gastrocnemius showed an increase in total MATR3 levels (~ 120 double, 90, 75, and 55 kDa combined) in mice ~ 1.5 months old transgenic mice from validation line 1579 compared to NT mice and mice from MATR3F115C lead line 1576 and 1573. Escape extension showed differences between b NT compared to c MATR3F115C transgenic mouse from validation line 1579 displaying a severe phenotype. Gross hindlimb muscle atrophy was apparent when comparing hindlimb of d NT to e MATR3F115C mice. H&E of f NT and g MATR3F115C where MATR3F115C gastrocnemius showed striking pathology including centralized nuclei (white arrow head), rounded fibers, smaller fibers, and subsarcolemmal vacuoles (white arrows). Immunohistochemistry of h NT gastrocnemius and i MATR3F115C showed that MATR3 immunoreactivity was elevated in the nucleus and cytoplasm of MATR3F115C mice. Panels b-e are from males. Scale bar measures 25 μm. (TIF 4722 kb) [file 40478_2018_631_MOESM2_ESM.tif]

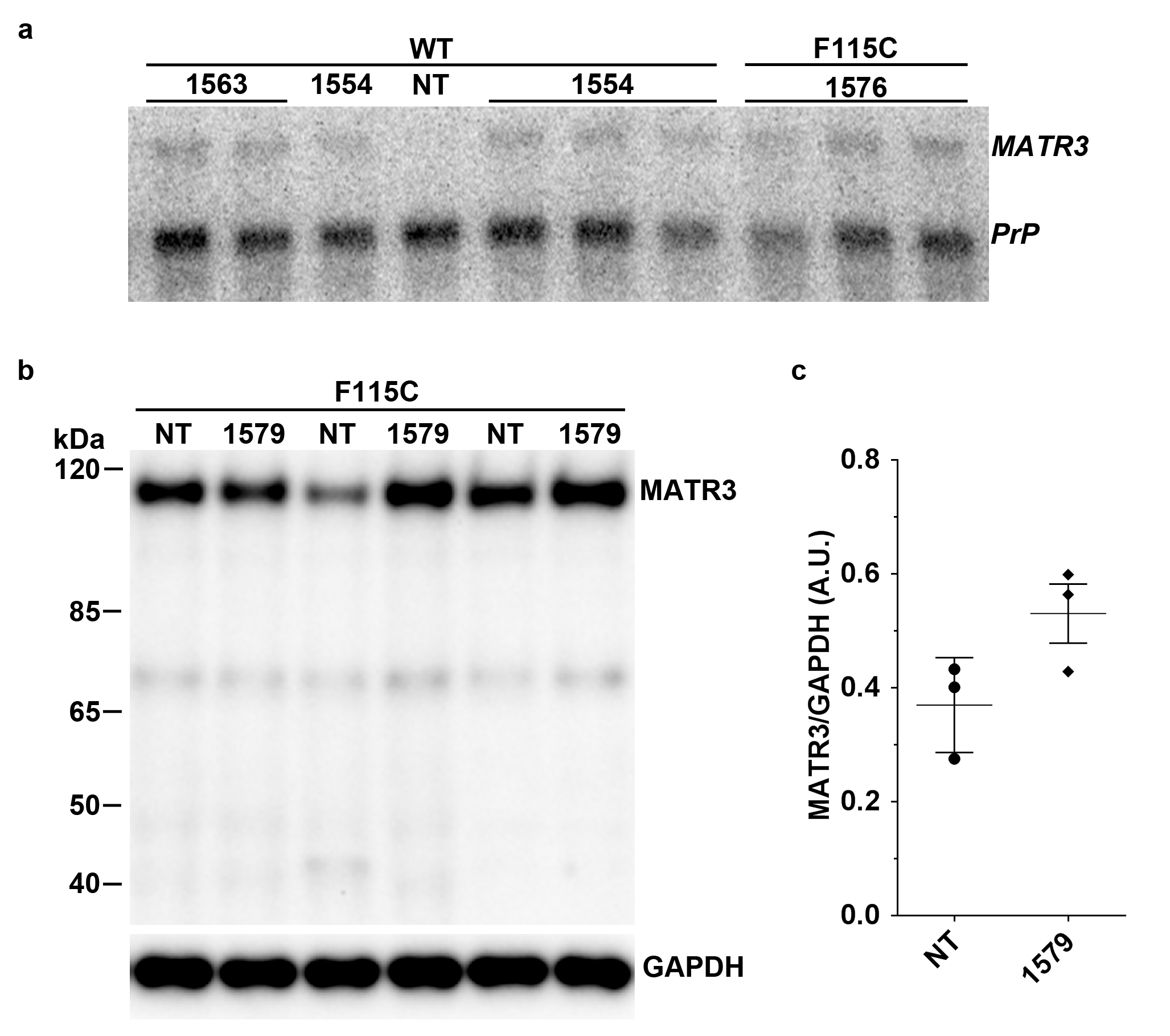

Supplement: Supplementary file 4 — Figure S2. MATR3 is not robustly elevated in the spinal cords of MATR3WT and MATR3F115C mice. a Northern blot analysis of cervical spinal cord showed limited transgenic MATR3 RNA expression in MATR3WT (lead line 1563 and validation line 1554) and MATR3F115C (lead line 1576) mice at ~ 4 months of age. Mouse prion (PrP) mRNA served as a loading control. b Western blot demonstrated that total MATR3 is not robustly elevated in spinal cord of MATR3F115C (validation line 1579) mice compared to NT mice at ~ 1.5 months of age. GAPDH was utilized as a loading control. c Quantification of Western blot showed no significant difference in total MATR3 levels in spinal cord of NT compared to MATR3F115C validation line 1579 mice (t-test, p > 0.05). (TIF 907 kb) [file 40478_2018_631_MOESM4_ESM.tif]

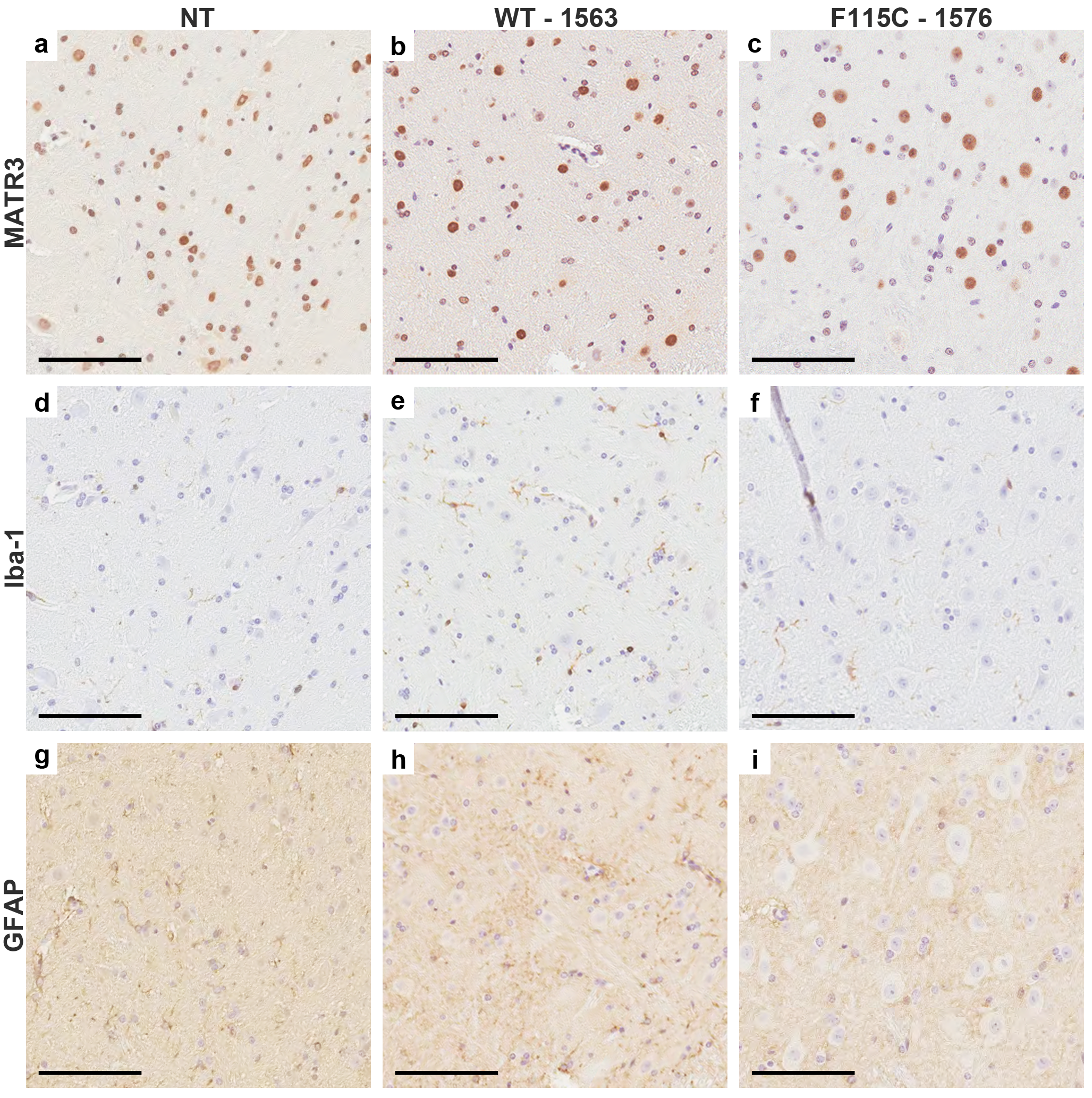

Supplement: Supplementary file 5 — Figure S3. Unremarkable spinal cord pathology in aged MATR3WT and MATR3F115C mice. MATR3 immunohistochemistry of spinal cord at ~ 10 months of age from a NT, b MATR3WT from lead line 1563, and c MATR3F115C from lead line 1576. Qualitatively, there appeared to be no difference in Iba-1 immunohistochemistry between d NT, e MATR3WT, and f MATR3F115C, or in GFAP immunohistochemistry of g NT, h MATR3WT, and i MATR3F115C spinal cords. Scale bar measures 100 μm. (TIF 6303 kb) [file 40478_2018_631_MOESM5_ESM.tif]
