# Supplementary material for: Analysis of spinal and muscle pathology in transgenic mice overexpressing wild-type and ALS-linked mutant MATR3
Source: Acta Neuropathol Commun. 2018 Dec 19;6:137. doi: 10.1186/s40478-018-0631-0 (PMC6299607; doi:10.1186/s40478-018-0631-0)
Supplement: Supplementary file 3 — Table S2. Average body weights for lead MATR3WT and MATR3F115C lines. MATR3WT mice (lead line 1563) were not significantly different when compared to age-matched NT males (~ 2 mo.: two-way ANOVA, p > 0.05; ~ 10 mo.: t-test, p > 0.05) and females (~ 2 mo.: two-way ANOVA, p > 0.05). At ~ 2 months, MATR3F115C mice (lead line 1576) weighed significantly less compared to age-matched and sex-matched NT mice (two-way ANOVA, p < 0.0001) and at ~ 10 months, male (two-way ANOVA, p < 0.0001) and female (two-way ANOVA, p < 0.05) MATR3F115C mice (lead line 1576) weighed significantly less compared to age- and sex-matched NT mice. Not significant, n.s; *, p ≤ 0.05; ****, p ≤ 0.0001. (DOCX 16 kb) [file 40478_2018_631_MOESM3_ESM.docx]

**Supplementary Table 2.** Average body weights for lead MATR3^WT^ and MATR3^F115C^ lines.

|  | Average Weight (g) | | | | | | | |
| --- | --- | --- | --- | --- | --- | --- | --- | --- |
| Founder Line | MATR3^WT^ 1563 | | | MATR3^F115C^ 1576 | | | | MATR3^F115C^ 1579 |
| Age | ~2 mo. | | ~10 mo. | ~2 mo. | | ~10 mo. | | ~1.3 mo. |
| Genotype | M | F | M | M | F | M | F | M |
| NT | 25.9 | 20.7 | 41.1 | 26.2 | 21.6 | 41.0 | 29.7 | 24.1 |
| Tg | 25.9 | 20.9 | 38.1 | 20.4 | 16.7 | 17.2 | 20.6 | 15.0 |
|  | n.s. | n.s | n.s | **** | **** | **** | * | **** |

MATR3^WT^ mice (lead line 1563) were not significantly different when compared to age-matched NT males (~2 mo.: two-way ANOVA, p>0.05; ~10 mo.: t-test, p>0.05) and females (~2 mo.: two-way ANOVA, p>0.05). At ~2 months, MATR3^F115C^ mice (lead line 1576) weighed significantly less compared to age-matched and sex-matched NT mice (two-way ANOVA, p<0.0001). However, by ~10 months, male (two-way ANOVA, p<0.0001) and female (two-way ANOVA, p<0.05) MATR3^F115C^ mice (lead line 1576) weighed significantly less compared to age- and sex-matched NT mice. Not significant, n.s; *, p≤0.05; ****, p≤0.0001.
